# Fully and semi-automated shape differentiation in **NGSolve**

**DOI:** 10.1007/s00158-020-02742-w

**Published:** 2020-11-05

**Authors:** Peter Gangl, Kevin Sturm, Michael Neunteufel, Joachim Schöberl

**Affiliations:** 1 TU Graz, Steyrergasse 30, 8010 Graz, Austria; 2 TU Wien, Wiedner Hauptstr. 8-10, 1040 Vienna, Austria

**Keywords:** Shape optimisation, Shape derivative, Automated differentiation, Shape Newton method

## Abstract

In this paper, we present a framework for automated shape differentiation in the finite element software NGSolve. Our approach combines the mathematical Lagrangian approach for differentiating PDE-constrained shape functions with the automated differentiation capabilities of NGSolve. The user can decide which degree of automatisation is required, thus allowing for either a more custom-like or black-box–like behaviour of the software. We discuss the automatic generation of first- and second-order shape derivatives for unconstrained model problems as well as for more realistic problems that are constrained by different types of partial differential equations. We consider linear as well as nonlinear problems and also problems which are posed on surfaces. In numerical experiments, we verify the accuracy of the computed derivatives via a Taylor test. Finally, we present first- and second-order shape optimisation algorithms and illustrate them for several numerical optimisation examples ranging from nonlinear elasticity to Maxwell’s equations.

**Supplementary Information:**

The online version contains supplementary material available at 10.1007/s00158-020-02742-w.

## Introduction

Numerical simulation and shape optimisation tools to solve the problems have become an integral part in the design process of many products. Starting out from an initial design, non-parametric shape optimisation techniques based on first- and second-order shape derivatives can assist in finding shapes of a product which are optimal with respect to a given objective function. Examples include the optimal design of aircrafts (Schmidt et al. 2013; 2011), optimal inductor design (Hömberg and Sokolowski 2003), optimisation of microlenses (Paganini et al. 2015), the optimal design of electric motors (Gangl et al. 2015), applications to mechanical engineering (Allaire et al. 2004; Laurain 2018), multiphysics problems (Feppon et al. 2019), or electrical impedance tomography (EIT) in medical sciences to name only a few (Hintermüller and Laurain 2008).

Shape optimisation algorithms are based on the concept of shape derivatives. Let $\mathcal P(\mathbf {R}^{d})$ denote the set of all subsets of **R**^*d*^. Furthermore, let $\mathcal A \subset \mathcal P(\mathbf {R}^{d})$ be a set of admissible shapes and $\mathcal {J}: \mathcal A \rightarrow \mathbf {R}$ be a shape function. Given an admissible shape $\varOmega \in \mathcal A$ and a sufficiently smooth vector field *V*, we define the perturbed domain *Ω*_*t*_ := (Id + *tV* )(*Ω*) for a small perturbation parameter *t* > 0. The shape derivative is defined as:

1 $$ \begin{array}{@{}rcl@{}} D \mathcal{J}(\varOmega)(V) \!:=\! \left. \left( \frac{d}{dt} \mathcal{J}(\varOmega_{t}) \right)\right\rvert_{t=0} = \underset{t \rightarrow 0}{\text{lim }} \frac{\mathcal{J}(\varOmega_{t}) - \mathcal{J}(\varOmega)}{t}. \end{array} $$

### *Remark 1*

We remark that a frequently used definition of shape differentiability is to require the mapping *V* ↦*J*((Id + *V* )(*Ω*)) being Fréchet differentiable in *V* = 0; see (Allaire 2007; Henrot and Pierre 2005; Murat and Simon 1976). This stronger notion of differentiability implies that the limit defined in (1) exists.

In most practically relevant applications, the objective functional depends on the shape of a (sub-)domain via the solution to a partial differential equation (PDE). Thus, one is facing a problem of PDE-constrained shape optimisation of the form:

2 $$ \begin{array}{ll} \qquad\qquad\qquad\underset{(\varOmega,u) \in \mathcal A\times Y }{\text{min }} J(\varOmega, u) \\ \text{s.t. } (\varOmega, u) \in \mathcal A\times Y : e(\varOmega; u,v) = 0 & \quad \text{ for all } v \in Y . \end{array} $$

Here, the second line represents the constraining boundary value problem posed on a Hilbert space *Y*, which we assume to be uniquely solvable for all admissible $\varOmega \in \mathcal A$. Denoting the unique solution for a given $\varOmega \in \mathcal A$ by *u*_*Ω*_, we introduce the notation for the reduced functional:

$$ \begin{array}{@{}rcl@{}} \mathcal{J}(\varOmega) := J(\varOmega,u_{\varOmega}). \end{array} $$

In order to be able to apply a shape optimisation algorithm to a given problem of this kind, the shape derivative (1) has to be computed; see the standard literature Delfour and Zolésio (2011a) and Sokołowski and Zolésio (1992) or Sturm (2015a) for an overview of different approaches. In the following, we focus on computing the so-called volume form of the shape derivative which in a finite element context is known to give a better approximation compared to the boundary form; see Hiptmair et al. (2015) and Berggren (2010).

The convergence of shape optimisation algorithms can be speeded up by using second-order shape derivatives. Given two sufficiently smooth vector fields *V*, *W*, and an admissible shape $\varOmega \in \mathcal A$, let *Ω*_*s*,*t*_ := (Id + *sV* + *tW*)(*Ω*) be the perturbed domain. Then, the second-order shape derivative is defined as:

3 $$ \begin{array}{@{}rcl@{}} D^{2} \mathcal{J}(\varOmega)(V)(W) := \left. \left( \frac{d^{2}}{dsdt} \mathcal{J}(\varOmega_{s,t}) \right)\right\rvert_{s,t=0}. \end{array} $$

Second-order information in Newton-type algorithms has been explored in the articles Novruzi and Roche (2000), Allaire et al. (2016), Paganini and Sturm (2019), Eppler et al. (2007), and Schulz (2014). Since the computation of second-order shape derivatives is more involved and error prone, several authors have employed automatic differentiation (AD) tools; see e.g. Schmidt (2018) and Ham et al. (2019) for two approaches based on the Unified Form Language (UFL) (Alnæs et al. 2014). In Ham et al. (2019), the authors present a fully automated shape differentiation software which uses the transformation properties on the finite element level. In Schmidt (2018) (see also the earlier work (Schmidt 2014)), the automated derivatives are computed using UFL. The strategies of Ham et al. (2019) and Schmidt (2018) differ in that, for the latter, the software computes an unsymmetric shape Hessian since it involves the term $D \mathcal {J}(\varOmega )(\partial VW)$. Optionally, the software allows to make the shape Hessian symmetric by requiring *∂V* *W* = 0. We will discuss the subtle difference and the relation between the two possible ways of defining shape Hessians in Remark 3 of Section 3.2. Let us also mention Dokken et al. (2020) where automated shape derivatives for transient PDEs in FEniCS and Firedrake are presented.

In this paper, we present an alternative framework for AD of PDE-constrained problems of type (2). There exist several approaches for the rigorous derivation of the shape derivative of PDE-constrained shape functionals; see Sturm (2015b) for an overview. The main idea, however, is always similar. After transforming the perturbed setting back to the original domain, shape differentiation in the direction of a given vector field reduces to the differentiation with respect to the scalar parameter *t* which now enters via the corresponding transformation and its gradient. It is shown in Sturm (2015a) that the shape derivative for a nonlinear PDE-constrained shape optimisation problem can be computed as the derivative of the Lagrangian with respect to the perturbation parameter. We will illustrate this systematic procedure for a number of different applications and utilise symbolic differentiation provided by the finite element software package NGSolve (Schöberl 2014) to obtain the shape derivative for different classes of PDE-constrained optimisation problems. NGSolve allows for the fast and efficient numerical solution of a large number of different boundary value problems. The aim of this paper is to extend NGSolve by the possibility of semi-automatic and fully automatic shape differentiation and optimisation.

Distinctly from previous approaches, we cover the following two points:

- A fully automated setting requiring as input the weak formulation of the constraint and the cost function,
- A semi-automated setting which offers a highly customisable user interface, but requires mathematical background knowledge.

### Structure of the paper

In Section 2, we give a brief introduction on how to solve a PDE in NGSolve and present its built-in auto-differentiation capabilities. The introduced syntax will also lay the foundation for the following sections. In Section 3, we present a first unconstrained shape optimisation problem and show how to solve it in NGSolve. For this purpose, we show how to compute the first- and second-order shape derivative in a semi-automated way. Section 4 extends the preceding section by incorporating a PDE constraint. The strategy is illustrated by means of a simple Poisson equation. We also show how to treat the computation of shape derivatives when the PDE is defined on surfaces. While the semi-automated shape differentiation presented in Sections 3 and 4 requires mathematical background knowledge, in Section 5 we show how the shape derivatives can be computed in a fully automated fashion. In the last section of the paper, we verify the computed formulas by a Taylor test, discuss optimisation algorithms and present several numerical optimisation examples including nonlinear elasticity, Maxwell’s equations and Helmholtz’s equation.

## A brief introduction to **NGSolve**

In this section, we give a brief overview of the main concepts of the finite element software NGSolve (Schöberl 2014). We first describe the main principles for numerically solving boundary value problems in NGSolve before focusing on its built-in automatic differentiation capabilities. In the subsequent sections of this paper, these ingredients will be combined to implement the shape derivative of unconstrained and PDE-constrained shape optimisation problems in an automated way.

### Solving PDEs with finite elements in **NGSolve**

In this section, we illustrate the syntax of NGSolve using the python programming language for the Poisson equation with homogeneous Dirichlet conditions as a model problem. We refer the reader to the online documentation

https://ngsolve.org/docu/latest/

for a more detailed description of the many features of this package.

Given a domain *Ω* ⊂**R**^*d*^ and a right-hand side *f*, we consider the model problem to find *u* satisfying:

$$ \begin{array}{@{}rcl@{}} - {\Delta} u = f &&\qquad \text{in } \varOmega, \\ u=0 &&\qquad\text{on } \partial \varOmega. \end{array} $$

The weak form of the model problem reads:

4 $$ \begin{array}{@{}rcl@{}} &&\text{Find } u \in H_0^1(\varOmega):  {\int}_\varOmega \nabla u \cdot \nabla w  \text{dx} \\ &&= {\int}_\varOmega f w  \text{dx} \quad \forall w \in H_0^1(\varOmega). \end{array} $$

We consider a ball of radius $\frac {1}{2}$ in two space dimensions centred at the point (0.5,0.5)^⊤^, i.e. *Ω* = *B*((0.5,0.5)^⊤^,0.5), and the right-hand side is defined by *f*(*x*_1_,*x*_2_) = 2*x*_2_(1 − *x*_2_) + 2*x*_1_(1 − *x*_1_). We will go through the steps for numerically solving this problem by the finite element method.

We begin by importing the necessary functionalities and setting up a finite element mesh. 
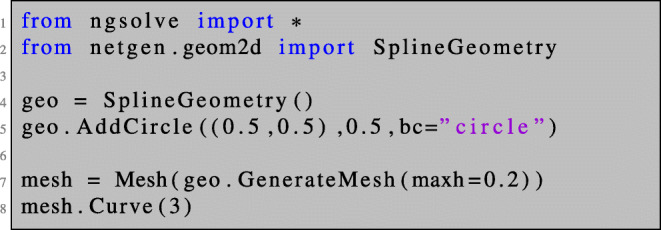


The first line imports all modules from the package NGSolve. The second line includes the SplineGeometry function which enables us to define a mesh via a geometric description, in our case a circle centred at (0.5,0.5)^⊤^ of radius 0.5. Finally, the mesh is generated in line 7, and in line 8 we specify that we want to use a curved finite element mesh for a more accurate approximation of the geometry. For that purpose, a projection-based interpolation procedure is used, see e.g. (Demkowicz 2004).

Next in line 9 we define an *H*^1^ conforming finite element space of polynomial degree 3 and include Dirichlet boundary conditions on the boundary of the domain *∂Ω* (referenced by the string ‘‘circle'' that we assigned in line 5). On this space, we define a trial function u in line 11 and a test function w in line 12. These are purely symbolic objects which are used to define boundary value problems in weak form. 
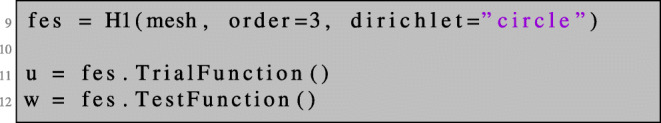


For a more compact presentation later on, we define a coefficient function X which combines the three spatial components: 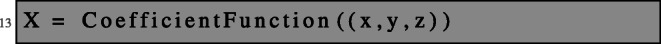


Now, the left- and right-hand sides of problem (4) can be conveniently defined as a bilinear or linear form, respectively, on the finite element space fes by the following lines. 
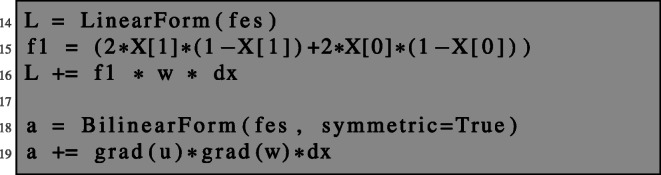


We assemble the system matrix coming from the bilinear form a and the load vector coming from L and solve the corresponding system of linear equations. 
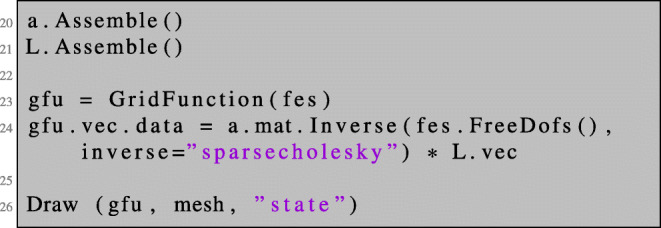


**Fig. 1:**
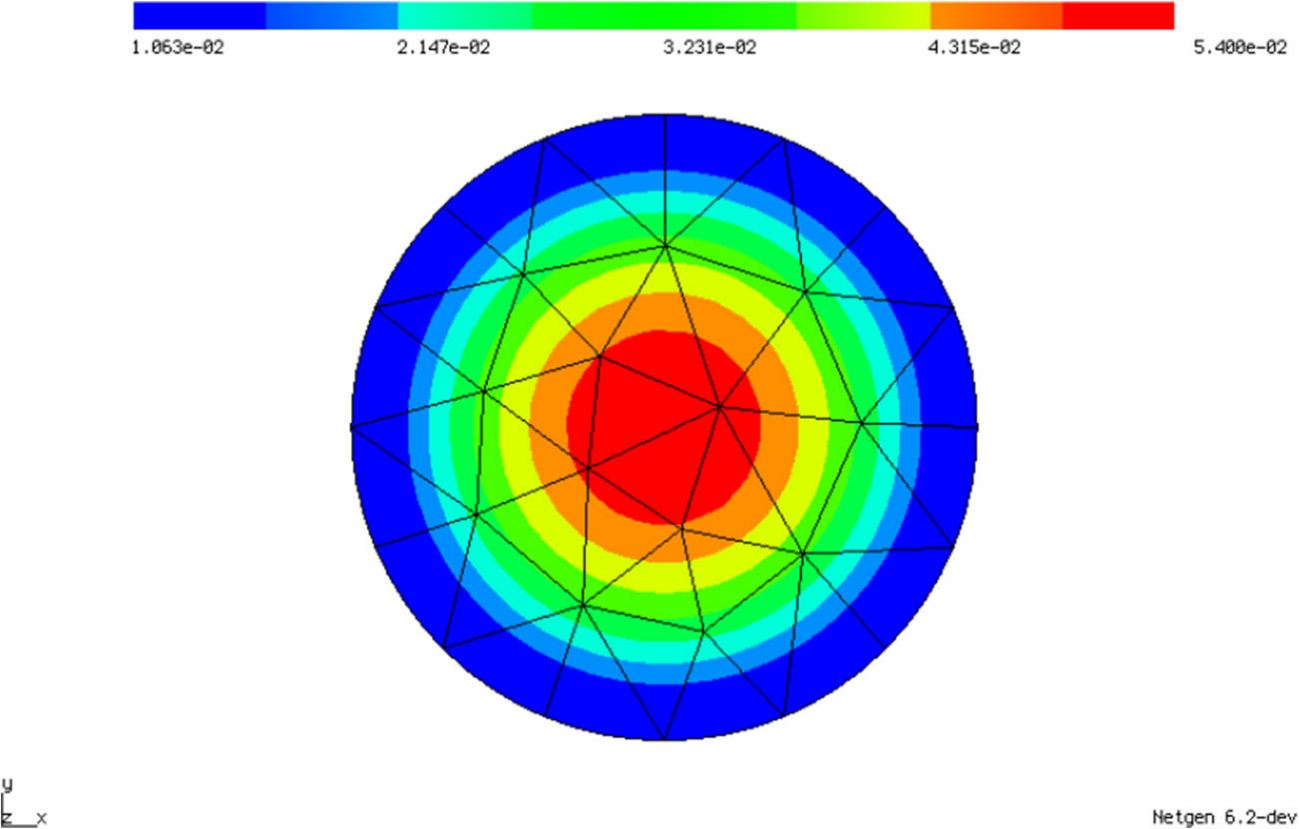
Solution of problem (4) by code fragments of Section 2.1 with 29 nodes, 40 (curved) triangular elements and polynomial order 3

Here, gfu is defined as a GridFunction over the finite element space fes. A GridFunction object is used to save the results by containing the corresponding finite element coefficient vectors. Furthermore, it can evaluate the stored finite element solution at a given mesh point. The Dirichlet conditions are incorporated into the direct solution of the linear system and the numerical solution is drawn in the graphical user interface. The numerical solution is depicted in Fig. 1.

### Automatic differentiation in **NGSolve**

**Fig. 2:**
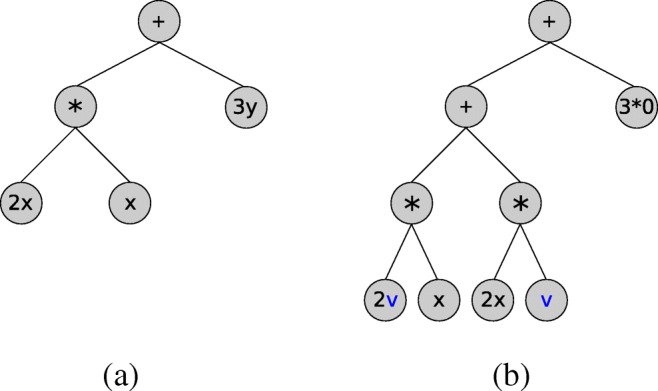
Illustration of Diff command for example expr= 2x*x + 3y. **a** Expression tree for *expr*. **b** Expression tree for expression obtained by call of expr.Diff(x, v)

In NGSolve, symbolic expressions are stored in expression trees; see Fig. 2 for an example. It is possible to differentiate an expression *expr* with respect to a variable *var* appearing in *expr* into a direction *dir* by the command

expr.Diff(var, dir).

Mathematically, this line corresponds to the directional derivative of g:=*expr* at *x* := *var* in direction *v* := *dir*, that is,

5 $$ Dg(x)(v). $$

When calling the Diff command for expr, the expression tree of *expr* is gone through node by node, and for each node the corresponding differentiation rules such as product rule or chain rule are applied. When a node represents the variable with respect to which the differentiation is carried out, it is replaced by the direction *dir* of differentiation.

Figure 2 shows the differentiation of the expression expr= 2x*x + 3y with respect to *x* into the direction given by *v*: 
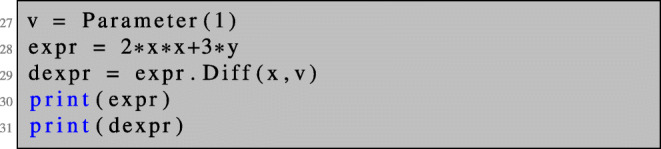


The output of print(expr) reads:


coef binary operation '+', real coef binary operation '*', real coef scale 2, real coef coordinate x, real coef coordinate x, real coef scale 3, real coef coordinate y, real

which translates to 2*x* ∗ *x* + 3*y* and corresponds to the expression tree depicted in Fig. 2a. The output of print(dexpr) reads:


coef binary operation '+', real coef binary operation '+', real coef binary operation '*', real coef scale 2, real coef N5ngfem28ParameterCoefficient FunctionE, real coef coordinate x, real coef binary operation '*', real coef scale 2, real coef coordinate x, real coef N5ngfem28ParameterCoefficient FunctionE, real coef scale 3, real coef 0, real

which translates to (2*v* ∗ *x* + 2*x* ∗ *v*) + 3 ∗ 0 and corresponds to the expression tree depicted in Fig. 2b. The coefficient

$$ \mathrm{\texttt{N5ngfem28ParameterCoefficientFunctionE}} $$

appearing therein is the C++ internal class name of the Python object Parameter.

NGSolve trial and test functions are purely symbolic objects used for defining bilinear and linear forms. Therefore, they do not depend on the spatial variables *x*, *y*, *z* as can be seen by differentiating them. NGSolve GridFunction s on the other hand represent functions in the finite element space. However, also for these objects, the space dependency is omitted when performing symbolic differentiation. The code segments 
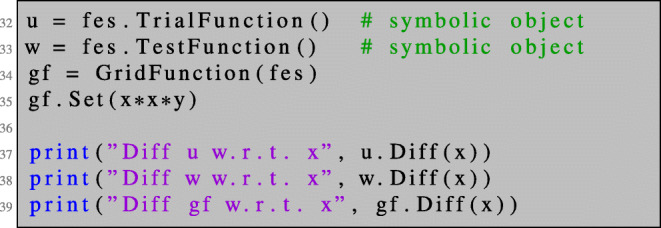


will give the following output:


Diff u w.r.t. x: ConstantCF, val = 0Diff w w.r.t. x: ConstantCF, val = 0Diff gf w.r.t. x: ConstantCF, val = 0

Here, the GridFunction.Set method takes a CoefficientFunction object and performs a (local) *L*^2^ best approximation into the underlying finite element space with respect to its natural norm and stores the resulting coefficient vector.

## Semi-automatic shape differentiation without constraints

We will illustrate the steps to be taken in order to obtain the shape derivative of a shape function in a semi-automatic way for a simple shape optimisation problem. For *Ω* ⊂**R**^*d*^ bounded and open and a continuously differentiable function *f* ∈ *C*^1^(**R**^*d*^), we consider the shape differentiation of the shape function:

6 $$ \begin{array}{@{}rcl@{}} \mathcal{J}(\varOmega) = {\int}_{\varOmega} f(x)  \text{dx}. \end{array} $$

Clearly, the minimiser of $\mathcal {J}$ over all measurable sets in **R**^*d*^ is given by *Ω*^∗^ = {*x* ∈**R**^*d*^ : *f*(*x*) < 0}. We also refer to Schiela and Ortiz (2017) for the computations of first- and second-order variations of functions of type (6) where *Ω* is a submanifold of **R**^*d*^.

### First-order shape derivative

Henceforth, we denote by *C*^0,1^(**R**^*d*^)^*d*^ the space of bounded and Lipschitz continuous vector fields *V* : **R**^*d*^ →**R**^*d*^. In view of Rademachers’ theorem (Evans 2010, Thm.6, p. 296), the space *C*^0,1^(**R**^*d*^)^*d*^ corresponds to the Sobolev space $W^{1,\infty }(\mathbf {R}^{d})^{d}$.

Given a vector field *V* ∈ *C*^0,1^(**R**^*d*^)^*d*^, we define the transformation:

$$ \begin{array}{@{}rcl@{}} T_{t}(x) := (\text{Id} + t  V)(x) , \quad x \in \mathbf{R}^{d},\quad t\ge 0. \end{array} $$

#### **Definition 1**

The first-order shape derivative of a shape function $\mathcal {J}$ at *Ω* in direction *V* ∈ *C*^0,1^(**R**^*d*^)^*d*^ is defined by:

7 $$ D\mathcal{J}(\varOmega)(V) = \lim_{t \rightarrow 0} \frac{\mathcal{J}(T_t(\varOmega)) - \mathcal{J}(\varOmega)}{t}. $$

#### Shape differentiation of unconstrained volume integrals

Using the transformation *y* = *T*_*t*_(*x*) and the notation *F*_*t*_ := *∂T*_*t*_ = *I* + *t∂V* for the Jacobian of the transformation *T*_*t*_, we get for $\mathcal {J}$ as in (6):

8 $$ \begin{array}{@{}rcl@{}} \mathcal{J}(\varOmega_{t}) ={} {\int}_{\varOmega_{t}}{} f(x^{\prime})  \text{dx}^{\prime} {}={} {\int}_{\varOmega} {}(f\circ T_{t})(x)  \det(F_{t}(x)) \mathrm{d}x. \end{array} $$

Now, let us explain how to compute the shape derivative of $\mathcal {J}$. Denoting

9 $$  G(T_t,F_t) := {\int}_\varOmega (f\circ T_t)(x) \det(F_t(x)) \mathrm{d}x, $$

the chain rule gives (formally)

10 $$ \begin{array}{@{}rcl@{}} \left. \frac{d}{dt} \mathcal{J}(\varOmega_{t}) \right|_{t=0} &= \left.\frac{d}{dt} G(T_{t},F_{t}) \right|_{t=0} \\ &= \left. \left( \frac{dG}{dT_{t}} \frac{d T_{t}}{dt} + \frac{dG}{dF_{t}} \frac{d F_{t}}{dt}\right)\right|_{t=0}. \end{array} $$

Using that $\frac {d T_{t}}{dt}(x) = V(x)$ and $\frac {d F_{t}}{dt}(x) = \partial V(x)$, we get for the shape derivative:

$$ \begin{array}{@{}rcl@{}} D \mathcal{J}(\varOmega)(V) = \left. \frac{d}{dt} \mathcal{J}(\varOmega_{t}) \right|_{t=0} = \left. \left( \frac{dG}{dT_{t}} V + \frac{dG}{dF_{t}} \partial V\right)\right|_{t=0}. \end{array} $$

This is the form we use for defining the first-order shape derivative in NGSolve. Note that a Lipschitz vector field is differentiable almost everywhere and hence *∂V* (*x*) is defined almost everywhere and bounded.

Given the function *f*(*x*_1_,*x*_2_) = (*x*_1_ − 0.5)^2^/*a*^2^ + (*x*_2_ − 0.5)^2^/*b*^2^ − *R*^2^ with *a* = 1.3, *b* = 1/*a* and *R* = 0.5, we implement the transformed cost function (8) as follows: 
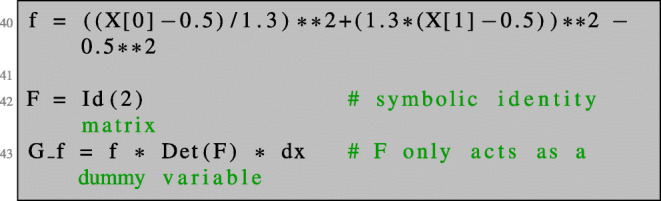


Here, we introduce the symbol F and assign to it the value of the identity matrix in line 42. This allows us to differentiate with respect to F. Then, we define the function *G* of (9) in line 43. The shape derivative is a bounded linear functional on a space of vector fields. We introduce a vector-valued finite element space VEC and define the object representing the shape derivative dJOmega_f as a linear functional on VEC. In line 48, we differentiate with respect to the spatial variables in the direction given by V. Note that X is the coefficient function we introduced in line 13. In line 49, we deal with the differentiation with respect to F.

##### *Remark 2*

Defining $\xi _{t} := \det (F_{t})$ and using $\frac {d}{dt}\xi _{t}|_{t=0} = \text {div} V$, it holds:

$$ \begin{array}{@{}rcl@{}} \left. \frac{dG}{d F_{t}} \frac{d F_{t}}{dt} \right|_{t=0} &= \left. \frac{d G}{d \xi_{t}} \frac{d \xi_{t}}{d F_{t}} \frac{d F_{t}}{dt} \right|_{t=0} = \left. \frac{d G}{d \xi_{t}} \frac{d \xi_{t}}{dt } \right|_{t=0} \\ &= \left.\frac{d G}{d \xi_{t}} \text{div}V \right|_{t=0} = {\int}_{\varOmega} f \text{div} V  \text{dx}. \end{array} $$

Therefore, we obtain for the first-order shape derivative the well-known formula:

11 $$ \begin{array}{@{}rcl@{}} D \mathcal{J}(\varOmega)(V) = {\int}_{\varOmega} \nabla f \cdot V + f  \text{div} V  \text{dx}. \end{array} $$

Finally, if *Ω* is smooth enough (for instance *C*^1^), it follows by integration by parts in (11) that the shape derivative is given by:

12 $$ D\mathcal{J}(\varOmega)(V) = {\int}_{\partial \varOmega} f V\cdot n \text{ds}, $$

where *n* denotes the outward pointing normal along *∂Ω*. 
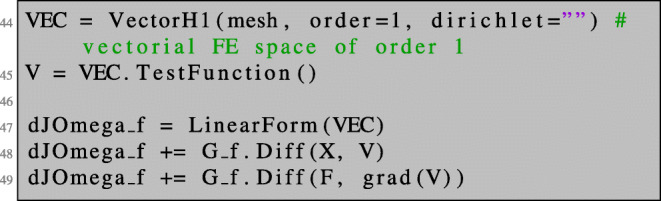


#### Shape differentiation of unconstrained boundary integrals

For *Ω* and *f* as in the previous section, we consider:

13 $$  \mathcal{J}_{bnd}(\varOmega) = {\int}_{\partial \varOmega} f(x)  \text{dx}. $$

Then, we get:

14 $$ \begin{array}{@{}rcl@{}} \mathcal{J}_{bnd}(\varOmega_{t}) &=& {\int}_{\partial \varOmega_{t}} f(x^{\prime})  \text{ds}_{x^{\prime}} \end{array} $$

15 $$ \begin{array}{@{}rcl@{}} & =& {\int}_{\partial \varOmega} (f\circ T_{t})(x)  \det(F_{t}(x)) | F_{t}(x)^{-\top} n(x) | \text{ds}_{x}, \end{array} $$

see e.g. (Sokołowski and Zolésio 1992, Prop. 2.47), with the outer unit normal vector *n* and |⋅| denoting the Euclidean norm. It is shown in (Sokołowski and Zolésio 1992, Prop. 2.50) that the shape derivative of (13) is given by:

$$ \begin{array}{@{}rcl@{}} D \mathcal{J}_{bnd}(\varOmega)(V) = {\int}_{\partial \varOmega} \nabla f \cdot V + f (\text{div}V - n^{\top} \partial V n) ds_{x}. \end{array} $$

Again, we can compute the shape derivative in NGSolve as the total derivative of expression (15) with respect to the parameter *t*. In NGSolve, the only difference lies in the necessity to use the trace of the gradient of a test vector field V. 
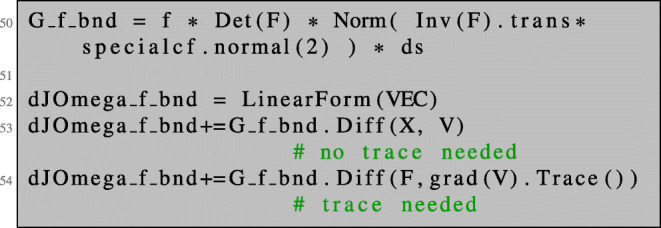


Note that the trace operator for gradients on the boundary is obligatory in NGSolve, whereas for direct evaluation of *H*^1^ trial and test functions itself it is optional.

### Second-order shape derivatives

For second-order shape derivatives, we consider perturbations of the form:

$$ \begin{array}{@{}rcl@{}} T_{s,t}(x) = (\text{Id} + s V + t W)(x), \quad x \in \mathbf{R}^{d}, \end{array} $$

for *s*,*t* ≥ 0 and define *Ω*_*s*,*t*_ := *T*_*s*,*t*_(*Ω*).

#### **Definition 2**

The second-order shape derivative of a shape function $\mathcal {J}$ at *Ω* in direction (*V*,*W*) ∈ *C*^0,1^(**R**^*d*^)^*d*^ × *C*^0,1^(**R**^*d*^)^*d*^ is defined by:

16 $$ D^{2}\mathcal{J}(\varOmega)(V)(W) =  \left. \frac{d^{2}}{dsdt} \mathcal{J}(\varOmega_{s,t}) \right\rvert_{s=t=0}. $$

#### *Remark 3*

We remark that if $\mathcal {J}$ is smooth enough, the second-order derivative as defined in (16) is symmetric by definition:

17 $$ D^{2}\mathcal{J}(\varOmega)(V)(W) = D^{2}\mathcal{J} (\varOmega)(W)(V). $$

We stress that this derivative is not the same as the shape derivative obtained by repeated shape differentiation, that is, it does not coincide with (see, e.g., (Delfour and Zolésio 2011b, Chap. 9, Sec. 6)):

18 $$  d^{2}\mathcal{J}(\varOmega)(V)(W) := \lim_{t \rightarrow 0}\frac{D\mathcal{J}(T_t^W(\varOmega))(V)- D\mathcal{J}(\varOmega)(V)}{t} $$

which is in general asymmetric.

The derivative defined in (18) is only symmetric if *∂V* *W* = 0 since it holds:

19 $$ d^{2}\mathcal{J}(\varOmega)(V)(W) = D^{2}\mathcal{J}(\varOmega)(V)(W) + D\mathcal{J}(\varOmega)(\partial VW), $$

see also the early work of Simon (1989) on this topic. However, in NGSolve, when repeating the shape differentiation procedure introduced in Section 3.1, we compute directly the second-order shape derivative as defined in (16). Here, we exploit the fact that trial functions are independent of the spatial coordinates; see also Section 2.2 and the example below.

Let us now exemplify the computation of the second-order shape derivative for the shape function $\mathcal {J}$ defined in (6). Similarly to the computations of the first derivative, we use the notation *F*_*s*,*t*_ := *∂T*_*s*,*t*_ = *I* + *s∂V* + *t∂W*. Then, we get:

$$ \begin{array}{@{}rcl@{}} \frac{d^{2}}{dsdt} \mathcal{J}(\varOmega_{s,t}) \left. \vphantom{\frac{d^{2}}{dsdt}} \right\rvert_{s=t=0} &=& \left. \frac{d^{2}}{dsdt} {\int}_{\varOmega_{s,t}} f(x) \mathrm{d}x \right\rvert_{s=t=0} \\ &=&\left. \frac{d^{2}}{dsdt} {\int}_{\varOmega} (f\circ T_{s,t})(x)  \det(F_{s,t}(x)) \mathrm{d}x \right\rvert_{s=t=0}. \end{array} $$

Again, using the notation

$$G(T_{s,t},F_{s,t}) = {\int}_{\varOmega} (f\circ T_{s,t})(x)  \det(F_{s,t}(x)) \mathrm{d}x,$$

we get

$$ \begin{array}{@{}rcl@{}} \left. \frac{d^{2}}{dsdt} \mathcal{J}(\varOmega_{s,t}) \right\rvert_{s=t=0} &=\left. \frac{d^{2}}{dsdt} G(T_{s,t},F_{s,t}) \right\rvert_{s=t=0}\\ &=\left. \frac{d}{ds} \left( \frac{dG}{dT_{s,t}} \frac{d T_{s,t}}{dt} + \frac{dG}{dF_{s,t}} \frac{d F_{s,t}}{dt} \right) \right\rvert_{s=t=0}. \end{array} $$

Using that $\frac {d^{2} T_{s,t} }{dsdt} = 0$ and $\frac {d^{2} F_{s,t} }{dsdt} = 0$, we get further:

20 $$ \begin{array}{@{}rcl@{}} \frac{d^{2}}{dsdt}& \mathcal{J}(\varOmega_{s,t}) \left. \vphantom{\frac{d^{2}}{dsdt}} \right\rvert_{s=t=0} \\ =&\left. \frac{d}{ds} \left( \frac{dG}{dT_{s,t}} \right) \frac{d T_{s,t}}{dt} + \frac{d}{ds}\left( \frac{dG}{dF_{s,t}} \right) \frac{d F_{s,t}}{dt} \right\rvert_{s=t=0} \\                   =& \left( \frac{d^{2} G}{d T_{s,t}^{2}} \frac{d T_{s,t}}{ds} + \frac{d^{2} G}{d F_{s,t} dT_{s,t}} \frac{d F_{s,t} }{d s} \right) \frac{d T_{s,t}}{dt} \\ &+ \left( \frac{d^{2} G}{d T_{s,t} dF_{s,t}} \frac{d T_{s,t} }{d s} + \frac{d^{2} G}{d F_{s,t}^{2}} \frac{d F_{s,t}}{ds} \right) \frac{d F_{s,t}}{dt} \left. \vphantom{\frac{d^{2}}{dsdt}} \right\rvert_{s=t=0}. \end{array} $$

Formula (20) is used for the automatic derivation of the second-order shape derivative in NGSolve. Using $\frac {d T_{s,t}}{ds}(x) = V(x)$, $\frac {d T_{s,t}}{dt}(x) = W(x)$ and $\frac {d F_{s,t}}{ds}(x) = \partial V(x)$, $\frac {d F_{s,t}}{dt}(x) = \partial W(x)$, we get:

21 $$ \begin{array}{@{}rcl@{}} \left. \frac{d^{2}}{dsdt} \mathcal{J}(\varOmega_{s,t}) \right\rvert_{s=t=0} &=& \left( \frac{d^{2} G}{d T_{s,t}^{2}} V + \frac{d^{2} G}{d F_{s,t} dT_{s,t}} \partial V \right) W \\ &+& \left( \frac{d^{2} G}{d T_{s,t} dF_{s,t}} V + \frac{d^{2} G}{d F_{s,t}^{2}} \partial V \right) \partial W \left. \vphantom{\frac{d^{2}}{dsdt}} \right\rvert_{s=t=0}. \end{array} $$

#### *Remark 4*

We remark that the formula (21) can be evaluated explicitly and reads:

$$ \begin{array}{@{}rcl@{}} D^{2}\mathcal{J}(\varOmega)(V,W) &=& {\int}_{\varOmega} \nabla^{2}f V \cdot W + \nabla f \cdot W  \text{div}V+ \nabla f \cdot V  \text{div}W \\ &+& f  \text{div}V  \text{div}W - f \partial V^{\top} : \partial W  \text{dx}. \end{array} $$

Formula (21) can be implemented in NGSolve as follows: 
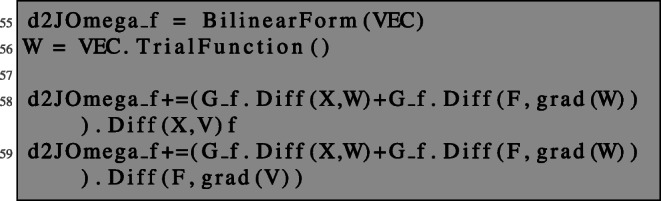


Notice that since W is a trial function, it is not affected by the differentiation with respect to X; see Section 2.2. Therefore, the terms coming from differentiating W with respect to the spatial coordinates X into the direction of V disappear and thus, although code lines 58–59 look like the “derivative of the derivative”, we actually compute formula (16) and not (18).

In the same fashion, second-order derivatives of boundary integrals of the form (13) can be computed. 
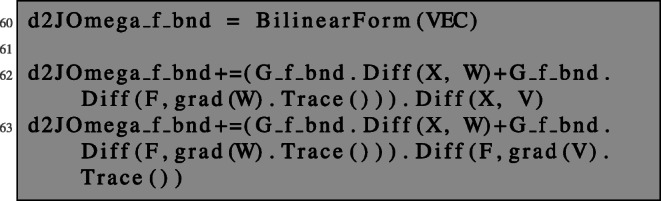


Again note that the trace operator is necessary when dealing with gradients on the boundary.

## Semi-automatic shape differentiation with PDE constraints

In this section, we describe the automatic computation of the shape derivative for the following type of equality-constrained shape optimisation problems:

22 $$  \min_{(\varOmega, u)} J(\varOmega, u) $$

subject to $(\varOmega ,u)\in \mathcal A\times Y $ solves

23 $$ e(\varOmega, u) = 0 , $$

where $e:\mathcal A\times Y \to Y^{*}$ with *e*(*Ω*,⋅) : *Y* (*Ω*) → *Y* (*Ω*)^∗^ represents an abstract PDE constraint with $ Y =\cup _{\varOmega \in \mathcal A}Y (\varOmega )$ being the union of Banach spaces *Y* (*Ω*) and $\mathcal A$ a set of admissible shapes. For any given $\varOmega \in \mathcal A$, we assume the PDE constraint (23) to admit a unique solution which we denote by *u*_*Ω*_. Moreover, let $\mathcal {J}(\varOmega ) := J(\varOmega , u_{\varOmega })$ denote the reduced cost functional. By introducing a Lagrangian function, we can henceforth deal with an unconstrained shape function $\mathcal L$ rather than a shape function $\mathcal {J}$ and a PDE constraint. We introduce the Lagrangian:

24 $$ \mathcal L(\varOmega, u, p) := J(\varOmega,u) + \langle e(\varOmega, u),p\rangle. $$

Now, an initial shape *Ω* is perturbed by a family of transformations *T*_*t*_, resulting in a new shape *Ω*_*t*_ := *T*_*t*_(*Ω*). Transforming back to the initial shape *Ω* leads to the Lagrangian:

25 $$  G(t,u,p) := \mathcal L(T_t(\varOmega),{\Phi}_t(u), {\Phi}_t(p)), \quad u,p\in Y (\varOmega), $$

where Φ_*t*_ : *Y* (*Ω*) → *Y* (*Ω*_*t*_) is a bijective mapping. Here, the transformation Φ_*t*_ depends on the differential operator involved. For instance:

- If $Y (\varOmega )={H^{1}_{0}}(\varOmega )$, then ${\Phi }_{t}(u) = u\circ T_{t}^{-1}$,
- If *Y* (*Ω*) = *H*(**curl**,*Ω*), then ${\Phi }_{t}(u) = \partial T_{t}^{-\top }(u\circ T_{t}^{-1})$,
- If *Y* (*Ω*) = *H*(div,*Ω*), then ${\Phi }_{t}(u) = \frac {1}{\det (\partial T_{t})} \partial T_{t} (u\circ T_{t}^{-1})$.

Intuitively, the transformations Φ_*t*_ are chosen in such a way that the transformed function Φ_*t*_(*u*) still belongs to the same space, but on a different domain. For the above three examples, this essentially requires to check how the differential operators ∇, **curl** and div transform under the change of variables *T*_*t*_, respectively. In fact, one can check that:

$$ \begin{array}{@{}rcl@{}} (\nabla u)\circ T_{t} & =& \partial T_{t}^{-\top} \nabla(u\circ T_{t}), \quad u\in {H^{1}_{0}}(\varOmega), \\ (\text{\textbf{curl}} u)\circ T_{t} & =& \frac{1}{\xi(t)} \partial T_{t} \text{\textbf{curl}}\left( \partial T_{t}^{\top} (u\circ T_{t}) \right), \quad u \in H(\text{\textbf{curl}},\varOmega),\\ (\text{div} u)\circ T_{t} & =& \frac{1}{\xi(t)} \text{div}\left( \xi(t) \partial T_{t}^{-1} (u\circ T_{t}) \right), \quad u\in H(\text{div},\varOmega), \end{array} $$

where $\xi (t):= \det (\partial T_{t})$; see also (Monk 2003, Section 3.9). The transformation rules are precisely given by the respective Φ_*t*_. We also note that for smooth functions this can be checked by direct computation.

Now the shape differentiability of (22–23) is reduced to proving that (see Sturm (2015b)):

26 $$ \begin{array}{@{}rcl@{}} D\mathcal{J}(\varOmega)(V)= \frac{d}{dt} G(t, u^{t}, 0)|_{t=0} = \partial_{t} G(0, u, p), \end{array} $$

where *u*^*t*^ := *u*_*t*_ ∘ *T*_*t*_ and *u*_*t*_ ∈ *Y* (*Ω*_*t*_) solves *e*(*Ω*_*t*_,*u*_*t*_) = 0 and *p* is the solution to the adjoint equation:

27 $$ p\in Y(\varOmega), \quad \partial_{u} G(0,u,p)(\varphi) =0 \quad \text{ for all } \varphi \in Y(\varOmega). $$

We stress that the choice of *p* as the solution of the adjoint equation is important in order for the second equality in (26) to hold. The verification of this equality depends on the specific PDE under consideration and can be accomplished by different methods. We refer the reader to Sturm (2015b) for an overview and remark that (26) holds for a large class of nonlinear PDE-constrained shape optimisation problems; see Sturm (2015a).

The rest of this section is organised as follows: We introduce a model problem, which is the minimisation of a tracking-type cost functional subject to Poisson’s equation in Section 4.1. We illustrate how the first- and second-order shape derivative for this PDE-constrained model problem can be obtained in NGSolve in Sections 4.2 and 4.3. Finally, we also briefly discuss the extension to partial differentiation equations on surfaces.

### PDE-constrained model problem

We will illustrate the derivation of the first- and second-order shape derivative for the minimisation of a tracking-type cost functional subject to Poisson’s equation on the unknown domain *Ω*. Let *d* = 2 or 3, $f, u_{d} \in H^{1}(\mathbf {R}^{d})$ and $\mathcal A \subset \mathcal P(\mathbf {R}^{d})$ be a set of admissible shapes. Here, $\mathcal P(\mathbf {R}^{d})$ denotes the power set of all subsets of **R**^*d*^. We consider the problem:

28a $$ \begin{array}{@{}rcl@{}} &\underset{(\varOmega,u) }{\text{min }} J(\varOmega, u) = {\int}_{\varOmega} |u-u_{d}|^{2}  \text{dx} \end{array} $$

subject to $(\varOmega ,u) \in \mathcal A \times {H^{1}_{0}}(\varOmega )$ solves

28b $$ \begin{array}{@{}rcl@{}} & \langle e(\varOmega,u),\psi\rangle := {\int}_{\varOmega} \nabla u \cdot \nabla \psi  \text{dx} - {\int}_{\varOmega} f \psi  \text{dx} = 0 \end{array} $$

for all $\psi \in {H_{0}^{1}}(\varOmega )$. The Lagrangian is given by:

29 $$ \mathcal L(\varOmega,\varphi,\psi) \!:=\! {\int}_\varOmega|\varphi - u_d|^2 \text{dx} + {\int}_{\varOmega} \nabla \varphi \cdot\nabla \psi  \text{dx} - {\int}_{\varOmega} f \psi \text{dx}. $$

Given an admissible shape *Ω*, a vector field *V* ∈ *C*^0,1^(**R**^*d*^)^*d*^ and *t* > 0 small, let *Ω*_*t*_ := (Id + *tV* )(*Ω*) be the perturbed domain. Therefore, the parametrised Lagrangian is given by:

30 $$ G(t,\varphi,\psi) \!:=\! \mathcal L(T_t(\varOmega) ,\varphi\circ T_t^{-1} ,\psi\circ T_t^{-1}), \quad \varphi,\psi\!\in\! H^1_0(\varOmega). $$

Changing variables yields:

31 $$ \begin{array}{@{}rcl@{}} G(t,\varphi, \psi) &=& {\int}_{\varOmega}|\varphi - {u_{d}^{t}}|^{2}  \det(F_{t}) \mathrm{d} x \\ &+& {\int}_{\varOmega} (F_{t}^{-\top}\nabla \varphi) \cdot (F_{t}^{-\top} \nabla \psi) \det(F_{t})  \text{dx} - {\int}_{\varOmega} f^{t} \psi  \\ &&\det(F_{t}) \text{dx} \\ &=:& \tilde G(T_{t}, F_{t}, \varphi, \psi), \end{array} $$

where ${u_{d}^{t}} = u_{d} \circ T_{t}$ and *f*^*t*^ = *f* ∘ *T*_*t*_. Here, we also transformed the gradient according to $(\nabla w)\circ T_{t} = F_{t}^{-\top } \nabla (w\circ T_{t})$ for $w \in {H^{1}_{0}}(\varOmega )$. Recall that, for a given $\varOmega \in \mathcal A$, *u*_*Ω*_ denotes the corresponding unique solution to (28b) and $\mathcal {J}(\varOmega )$ the reduced cost functional, $\mathcal {J}(\varOmega ) := J(\varOmega , u_{\varOmega })$. Let $u^{t} \in {H_{0}^{1}}(\varOmega )$ be the solution of the perturbed state equation brought back to the original domain *Ω*, that is, $u^{t} \in {H_{0}^{1}}(\varOmega )$ is the unique solution to:

32 $$ \begin{array}{@{}rcl@{}} \partial_{\psi} G(t, u^{t}, 0)(\psi) = 0 \quad \text{ for all } \psi \in {H_{0}^{1}}(\varOmega). \end{array} $$

Note that, for *u*^*t*^ defined by (32), it holds $\mathcal {J}(\varOmega _{t}) = G(t, u^{t}, \psi )$ for all $\psi \in {H_{0}^{1}}(\varOmega )$ and therefore also $D\mathcal {J}(\varOmega )(V) = \frac {d}{dt} G(t, u^{t}, \psi ) $ for all $\psi \in {H_{0}^{1}}(\varOmega )$.

It can easily be shown that (26) holds and thus the shape derivative in the direction of a vector field *V* ∈ *C*^0,1^(**R**)^*d*^ is given by:

$$D \mathcal{J}(\varOmega)(V) = \partial_{t} G(0, u, p), $$

where $p \in {H_{0}^{1}}(\varOmega )$ denotes the adjoint state and is defined as the unique solution $p \in {H_{0}^{1}}(\varOmega )$ to

33 $$ \begin{array}{@{}rcl@{}} \partial_{\varphi} G(0, u, p)(\hat \varphi) = 0 \quad \text{ for all } \hat \varphi \in {H_{0}^{1}}(\varOmega), \end{array} $$

or explicitly

34 $$ \begin{array}{@{}rcl@{}} {\int}_{\varOmega} \nabla \hat \varphi \cdot \nabla p  \text{dx} = - 2 {\int}_{\varOmega} (u-u_{d}) \hat \varphi  \text{dx} \quad \text{ for all } \hat \varphi \in {H_{0}^{1}}(\varOmega). \end{array} $$

### First-order shape derivative

By the discussion above, the first-order shape derivative is given by *∂*_*t*_*G*(0,*u*,*p*) with *G* defined in (31) and *u* and *p* the unique solutions to the boundary value problems (28b) and (34), respectively.

Writing $\tilde G(T_{t}, F_{t}) := \tilde G(T_{t}, F_{t}, u, p) = G(t,u,p), $ we obtain in analogy to the unconstrained problem:

$$ \begin{array}{@{}rcl@{}} D \mathcal{J}(\varOmega)(V) = \left. \frac{d}{dt} \mathcal{J}(\varOmega_{t}) \right|_{t=0} = \left. \left( \frac{d \tilde G}{dT_{t}} V + \frac{d \tilde G}{dF_{t}}\partial V \right)\right|_{t=0}. \end{array} $$

We can compute explicitly:

35 $$ \begin{array}{@{}rcl@{}} \frac{d \tilde G}{dF_{t}} |_{t=0} \partial V &=& {\int}_{\varOmega} \text{div}(V)(u-u_{d})^{2} - (\partial V + \partial V^{\top})\nabla u\cdot \nabla p\\ &-& \text{div}(V) \nabla u\cdot \nabla p - fp \text{div}(V) \text{dx}, \end{array} $$

36 $$ \begin{array}{@{}rcl@{}} \frac{d \tilde{G}}{dT_{t}} |_{t=0}V &=& {\int}_{\varOmega} - 2(u-u_{d}) \nabla u_{d}\cdot V - \nabla f\cdot V p \text{dx}. \end{array} $$

Now, we are in a position to compute the first-order shape derivative for the PDE-constrained shape optimisation problem (28) in NGSolve. After solving the state equation as shown in Section 2.1, the adjoint equation can be solved as follows. 
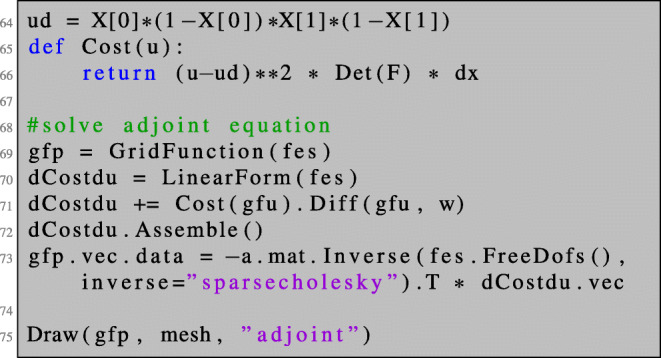


We can now define the Lagrangian (31) such that the shape derivative can be obtained by the same procedure as in the unconstrained setting. Note that lines 82–83 coincide with lines 48–49. 
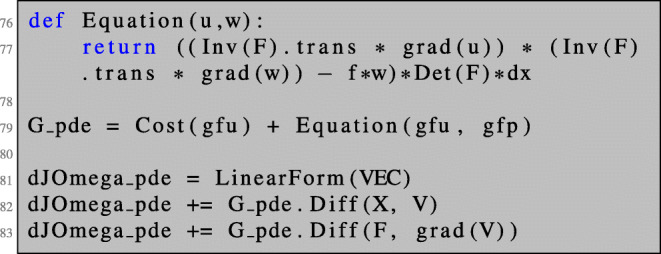


### Second-order shape derivative

Let us introduce the notation:

37 $$ \begin{array}{@{}rcl@{}} \langle E_{V,W}(s,t)\varphi,\psi\rangle &:=& {\int}_{\varOmega} (F_{s,t}^{-\top}\nabla \varphi) \cdot (F_{s,t}^{-\top} \nabla \psi) \det(F_{s,t})  \text{dx}\\ &-& {\int}_{\varOmega} f\circ T_{s,t} \psi  \det(F_{s,t}) \text{dx} \end{array} $$

38 $$ \begin{array}{@{}rcl@{}} J_{V,W}(s,t;\varphi)&:= &{\int}_{\varOmega}|\varphi - u_{d}\circ T_{s,t} |^{2}  \det(F_{s,t}) \mathrm{d} x \end{array} $$

and

39 $$ \begin{array}{@{}rcl@{}} G_{V,W}(s,t,u, p):=& \langle E_{V,W}(s,t)u,p\rangle + J_{V,W}(s,t;u), \end{array} $$

where *T*_*s*,*t*_(*x*) = *x* + *sV* (*x*) + *tW*(*x*) and *F*_*s*,*t*_ := *∂T*_*s*,*t*_. We observe that

40 $$ \mathcal{J}(T_{s,t}(\varOmega)) = G_{V,W}(s,t,u^{s,t}, p^{s,t}) $$

with $(u^{s,t},p^{s,t})\in {H^{1}_{0}}(\varOmega )\times {H^{1}_{0}}(\varOmega )$ being the solution to

41 $$ \begin{array}{@{}rcl@{}} \partial_{p} G_{V,W}(s,t,u^{s,t},0)(\varphi) & = 0 \quad \text{ for all } \varphi\in {H^{1}_{0}}(\varOmega), \end{array} $$

42 $$ \begin{array}{@{}rcl@{}} \partial_{u} G_{V,W}(s,t,u^{s,t},p^{s,t})(\psi) & = 0 \quad \text{ for all } \psi\in {H^{1}_{0}}(\varOmega) \end{array} $$

for *s*,*t* ≥ 0. In case, *t* = 0 we write *u*^*s*^ := *u*^*s*,*t*^|_*t*= 0_ and *p*^*s*^ := *p*^*s*,*t*^|_*t*= 0_ and similarly for *t* = *s* = 0 we write *u* := *u*^*s*,*t*^|_*s*=*t*= 0_ and *p* := *p*^*s*,*t*^|_*s*=*t*= 0_. Therefore, consecutive differentiation of (40) first with respect to *t* at 0 and then with respect to *s* at 0 yields:

43 $$ \begin{array}{@{}rcl@{}} &&D^{2} \mathcal{J}(\varOmega)(V)(W) = \frac{d^{2}}{dsdt} G_{V,W}(s,t,u^{s,t},p^{s,t})|_{s=t=0}\\ &&= \frac{d}{ds}\partial_{t} G_{V,W}(s,0,u^{s},p^{s})|_{s=0}\\ &&= \partial_{s}\partial_{t} G_{V,W}(0,0,u,p) + \partial_{u} \partial_{t} G_{V,W}(0,0,u,p)(\partial_{s} u^{0})\\ &&+ \partial_{p} \partial_{t} G_{V,W}(0,0,u,p)(\partial_{s} p^{0}), \end{array} $$

where $\partial _{s} u^{0} \in {H^{1}_{0}}(\varOmega )$ solves the material derivative equation:

44 $$ \partial_u\partial_p G_{V,W}(0,0,u,0)(\psi)(\partial_s u^0) = - \partial_s\partial_p G_{V,W}(0,0,u,0)(\psi) $$

for all $\psi \in {H^{1}_{0}}(\varOmega )$ or, equivalently

45 $$ \langle \partial_u E_{V,W}(0,0)(\partial_s u^0),\psi\rangle = - \langle \partial_s E_{V,W}(0,0)u,\psi\rangle $$

for all $\psi \in {H^{1}_{0}}(\varOmega )$. Note that (45) is obtained by differentiating (41) with respect to *s* and setting *s* = *t* = 0. Similarly, the function $\partial _{s} p^{0} \in {H^{1}_{0}}(\varOmega )$ solves the material derivative equation obtained by differentiating (42) with respect to *s* for *s* = *t* = 0,

46 $$ \begin{array}{@{}rcl@{}} \partial_{p}\partial_{u} G_{V,W}(0,0,u,p)(\psi)(\partial_{s} p^{0}) &=& - {\partial_{u}^{2}} G_{V,W}(0,0,u,p)(\psi)(\partial_{s} u^{0}) \\ &-& \partial_{s}\partial_{u} G_{V,W}(0,0,u,p)(\psi) \end{array} $$

for all $\psi \in {H^{1}_{0}}(\varOmega )$. The introduction of the adjoint variable *p* is analogous to the computation of the first-order shape derivative. However, in contrast to the first-order derivative, the evaluation of $D^{2} \mathcal {J}(\varOmega )(V)(W)$ requires the computation of the material derivatives *∂*_*s*_*u*^0^ and *∂*_*s*_*p*^0^.

Formally, (44) and (46) can be written as an operator equation with *x* = (0,0,*u*,*p*):

47 $$ \begin{pmatrix} \partial_u^2 G_{V,W}(x) & \partial_p\partial_u G_{V,W}(x) \\ \partial_u\partial_p G_{V,W}(x) & 0 \end{pmatrix} \begin{pmatrix} \partial_s u^0 \\ \partial_s p^0 \end{pmatrix} = -\begin{pmatrix} \partial_s\partial_u G_{V,W}(x) \\ \partial_s\partial_p G_{V,W}(x) \end{pmatrix}. $$

So to evaluate the second derivative (43) in some direction (*V*,*W*), we have to solve the system (47).

This is realised in NGSolve by setting up a combined finite element space which we denote by X2. We define trial and test functions as well as grid functions representing the deformation vector fields *V* and *W*, which we initialise with some functions. 
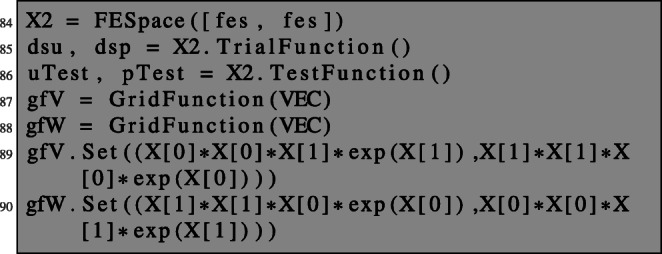


We define a 2×2 block bilinear form as well as a 2×1 block linear form which will represent the left- and right-hand sides of (47), respectively. The operator equation in (47) can be conveniently defined by differentiating the Lagrangian with respect to the corresponding variables. 
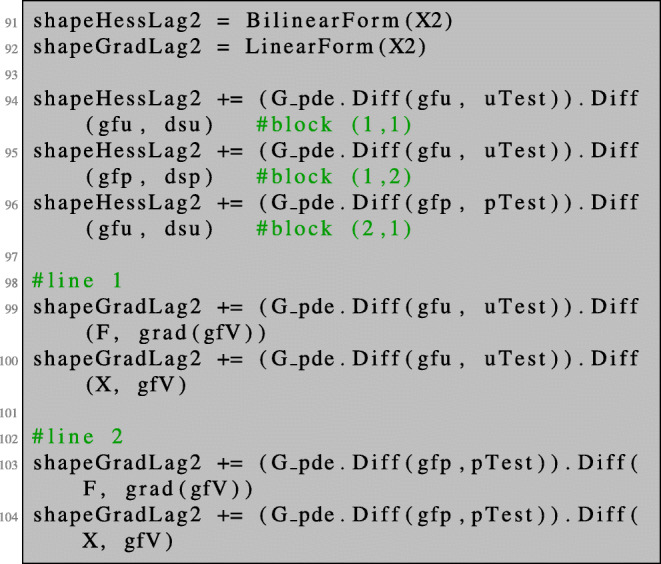


We can solve this combined system for *∂*_*s*_*u*^0^ and *∂*_*s*_*p*^0^ and access and visualise the two components in the following way: 
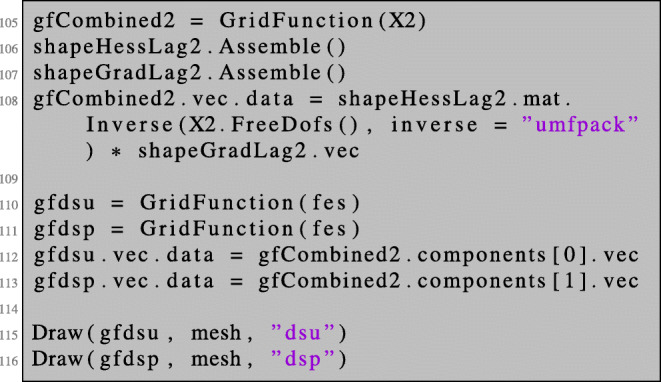


In order to obtain the second-order shape derivative in the direction given by (*V*,*W*), it remains to evaluate the term (43). We define the three terms of (43) as bilinear forms, assemble them, and perform vector-matrix-vector multiplications: 
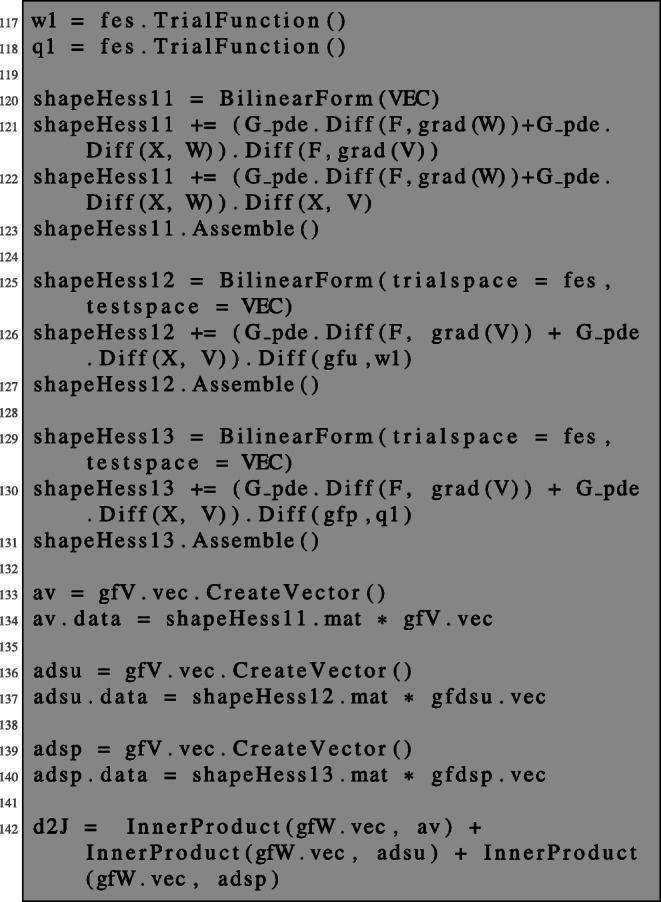


### PDEs on surfaces

The automated shape differentiation is not restricted to partial differential equations on domains *Ω*, but is readily extended to surface PDEs. We consider a two-dimensional closed surface *M* ⊂**R**^3^ and denote by *n* the normal field along *M*. Let $u_{d}\in H^{1}(\mathbf {R}^{3})$ be given and define:

48 $$  J(M,u) = {\int}_M |u-u_d|^2 \text{ds}, $$

where *u* ∈ *H*^1^(*M*) solves the surface equation

49 $$  {\int}_M \nabla^M u\cdot \nabla^M \psi + u \psi \text{ds} =\! {\int}_M f \psi \text{ds} \quad \text{ for all } \psi\! \in\! H^1(M), $$

where ∇^*M*^*ψ* denotes the tangential gradient of *ψ*; see (Delfour and Zolésio 2011b, p. 493, Def.5.1). We assume that the function *f* ∈ *H*^1^(**R**^3^) is given. The Lagrangian is given by:

$$ \begin{array}{@{}rcl@{}} \mathcal L(M,\varphi,\psi) &:=& {\int}_{M} |\varphi - u_{d}|^{2} \text{ds} + {\int}_{M} \nabla^{M} \varphi \cdot\nabla^{M} \psi + \varphi\psi \text{ds} \\ &-& {\int}_{M} f \psi  \text{ds}. \end{array} $$

As in the previous section, we fix an admissible shape *M* and let *M*_*t*_ := (Id + *tV* )(*M*) be a small perturbation of *M* by means of a vector field *V* ∈ *C*^1^(**R**^3^)^3^ for *t* > 0 small. The parametrised Lagrangian is given by:

50 $$ G(t,\varphi,\psi) \!:=\! \mathcal L(T_t(M) ,\varphi\circ T_t^{-1}, \psi\circ T_t^{-1}), \quad \varphi,\psi\!\in\! H^1(M\!). $$

Define the density $\omega (F_{t}) := \det (F_{t}) |F_{t}^{-\top }n|$. Changing variables and using

51 $$ \begin{array}{@{}rcl@{}} (\nabla^{M_{t}}\varphi)\circ T_{t} =& B(F_{t})\nabla^{M}(\varphi\circ T_{t}), \\ B(F_{t}) =& \left( I-\frac{F_{t}^{-\top}n}{|F_{t}^{-\top}n|}\otimes \frac{F_{t}^{-\top}n}{|F_{t}^{-\top}n|}\right) F_{t}^{-\top}, \end{array} $$

yields

52 $$ \begin{array}{@{}rcl@{}} G(t,\varphi, \psi) &=& {\int}_{M}|\varphi - {u_{d}^{t}}|^{2}  \omega(F_{t}) \text{ds} \\ &+& {\int}_{M} \left( (B(F_{t})\nabla \varphi) \cdot (B(F_{t}) \nabla \psi) + \varphi\psi \right) \omega(F_{t}) \text{ds} \\ &-& {\int}_{M} f^{t} \psi \omega(F_{t}) \text{ds}, \end{array} $$

where ${u_{d}^{t}} = u_{d} \circ T_{t}$ and *f*^*t*^ = *f* ∘ *T*_*t*_.

Writing $\tilde G(T_{t}, F_{t}) := G(t,u,p)$, we obtain in analogy to the domain case:

53 $$ \begin{array}{@{}rcl@{}} D \mathcal{J}(\varOmega)(V) = \left. \left( \frac{d \tilde G}{dT_{t}} V + \frac{d \tilde G}{dF_{t}} \partial V\right)\right|_{t=0}. \end{array} $$

We can compute explicitly:

54 $$ \begin{array}{@{}rcl@{}} \frac{d \tilde G}{dF_{t}}|_{t=0}V &=& {\int}_{M} \text{div}^{M}(V)(u-u_{d})^{2}\\ &&- (\partial^{M} V + \partial^{M} V^{\top})\nabla^{M} u\cdot \nabla^{M} p\\ &&+ \text{div}^{M}(V) (\nabla^{M} u\cdot \nabla^{M} p + up)\\ &&- fp \text{div}^{M}(V) \text{ds}, \end{array} $$

55 $$ \begin{array}{@{}rcl@{}} \frac{d \tilde G}{dT_{t}} |_{t=0} \partial V & = & {\int}_{M} - 2(u-u_{d}) \nabla u_{d}\cdot V - \nabla f\cdot V p \text{ds}, \end{array} $$

where *∂*^*M*^*V* denotes the tangential Jacobian of *V* defined by (*∂*^*M*^*V* )_*ij*_ := (∇^*M*^*V*_*i*_)_*j*_ for $i,j=1,\dots ,d$, and div^*M*^(*V* ) := *∂*^*M*^*V* : *I* the tangential divergence, which is defined as the trace of the tangential Jacobian; see (Delfour and Zolésio 2011b, p. 495).

The implementation is analogous to the previous sections. We will only illustrate first-order derivatives here. We first define the geometry of the unit sphere, create a surface mesh, and define a finite element space on the surface mesh: 
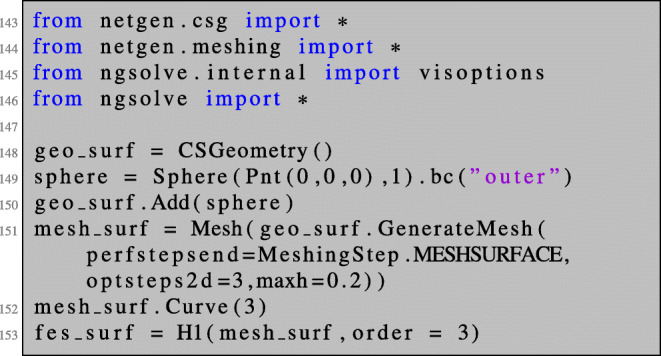


Next, we define the transformed cost function and partial differential equation needed for setting up the Lagrangian (52). Here, we again make use of a symbolic object F to which we assign the identity matrix. We define the tangential determinant *ω* and the matrix *B* defined in (51) as functions of the deformation gradient *F*_*t*_. 
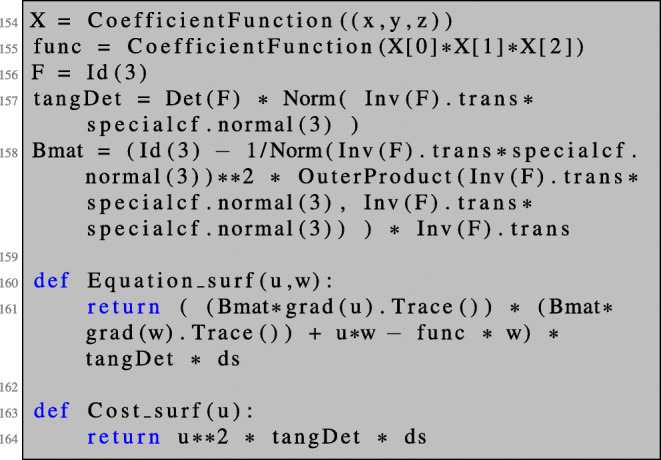


Now, we can define the bilinear form and solve the state equation. Here, the right-hand side of the equation is included in the bilinear form and the boundary value problem—although linear—is solved by Newton’s method (which terminates after only one iteration) for convenience. 
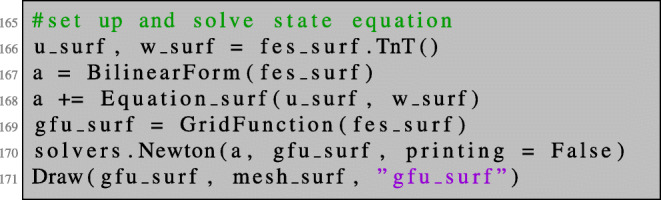


Using Newton’s method for solving the linear boundary value problem allows us to define both the left- and right-hand sides of the PDE using only one BilinearForm a (which, strictly speaking, is not bilinear anymore). This way, we can reuse Equation_surf as defined in lines 160–161 to define the boundary value problem in line 168.

The adjoint equation is solved as usual: 
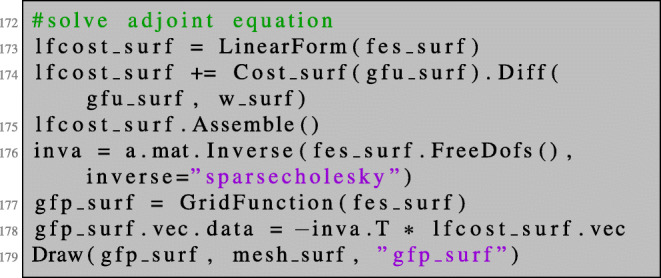


The shape derivative is obtained as in the case of PDEs posed on volumes by the evaluation of (53): 
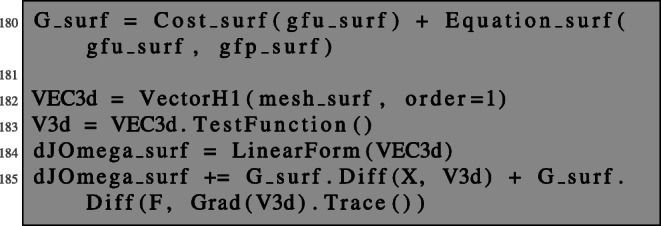


## Fully automated shape differentiation

In the previous sections, we used the automatic differentiation capabilities of NGSolve to alleviate the shape differentiation procedure. However, so far, we still had to include some knowledge about the problems at hand. So far, it was necessary to define the objective function or Lagrangian *G* in the correct way, accounting for the correct transformation rules between perturbed and unperturbed domains. In this section, we will show that also this step can be automated since all necessary information are already included in the functional setting. The fully automated shape differentiation is incorporated by the command:

DiffShape(...).

In particular, in the fully automated setting, it is enough to set up the cost function or Lagrangian for the unperturbed setting. For a shape function of the type (6), we can define the shape derivative of the cost function in the following way: 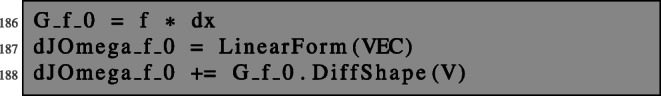 Note that there is no term of the form Det(F) showing up in line 186. Here, the transformation of the domain is taken care of automatically. It can be checked that this really gives the same result as dJOmega_f defined in lines 48–49. 
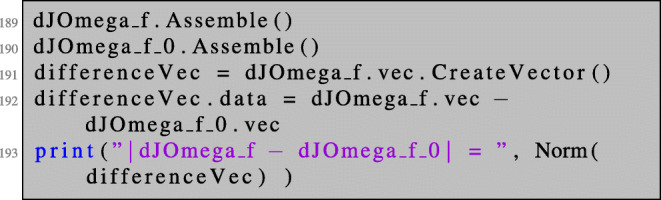


The above code gives the output:


|dJOmega_f - dJOmega_f_0| =1.571008573810619e-17

which confirms our claim. The same holds true for second-order shape derivatives. The lines 58–59 can be replaced by a repeated call of DiffShape(...): 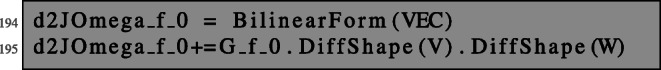


Again, it can be verified that d2JOmega_f_0 coincides with the previously defined quantity d2JOmega_f. Note that slightly different results may occur due to different integration rules used. This can be cured by enforcing an integration rule of higher order for G_f, i.e. by replacing the symbol dx in the definition of G_f with dx(bonus_intorder= 2).

In the more general setting of PDE-constrained shape optimisation, the procedure is very similar. Here, the idea exploited in the implementation of the command DiffShape(...) is to just differentiate the general expression (25) with respect to the parameter *t*. The transformations Φ_*t*_ appearing in (25), which depend on the functional setting of the PDE, are identified automatically from the finite element space from which the corresponding functions originate. The shape derivative of lines 82–83 can be obtained by the following code. 
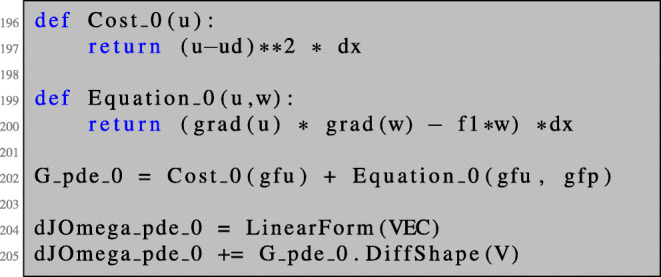


Here, gfu and gfp represent the solutions to the state and adjoint equations, respectively, and must have been computed previously. The bilinear form shapeHess11 used in Section 4.3 (see lines 121–122) can be obtained similarly: 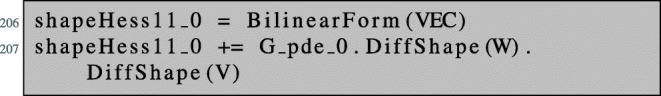


The same holds true for boundary integrals 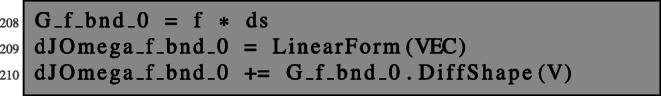


and surface PDEs 
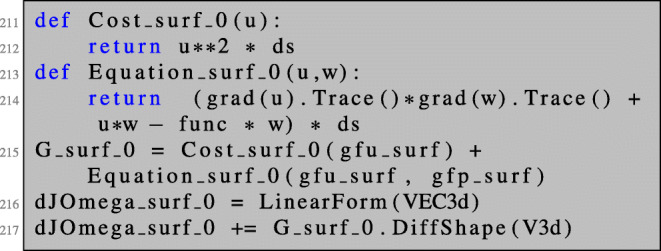


as well as their respective second-order derivatives.

### *Remark 5*

We remark that the fully automated differentiation using DiffShape(...) should be seen to complement the semi-automated shape differentiation techniques introduced in Sections 3 and 4 rather than to replace them. Using the semi-automated differentiation, the user has the possibility to, on the one hand, keep control over the involved terms, and on the other hand also to adjust the shape differentiation to their custom problems which may be non-standard. As an example where the semi-automated differentiation may be beneficial compared with the fully automated differentiation, we mention the case of time-dependent PDE constraints considered in a space-time setting when a shape deformation is only desired in the spatial coordinates; see Section 7.8. Of course, when one is interested in the shape derivative for a more standard problem, the fully automated way appears to be more convenient and less error prone.

### *Remark 6*

We have seen that the command DiffShape(…) allows computing the shape derivative of unconstrained shape optimisation problems in a fully automated way without specifying any transformation rules; see line 188. For the practically more relevant case of PDE-constrained shape optimisation problems, the state and adjoint equations have to be solved beforehand also in the fully automated context using DiffShape(…). We remark that this can be easily achieved by defining a custom function solvePDE() as it is done for the case of a linear PDE in lines 227–234. Since the purpose of this paper is to illustrate a convenient way of computing shape derivatives and performing shape optimisation rather than to provide a tool for black-box optimisation, this step is left to the user and is not automated, leaving more freedom in the choice of e.g. solvers for the arising linear systems.

## Optimisation algorithms

In this section, we discuss how to use optimisation algorithms in conjunction with the automated shape differentiation explained in the previous sections. The starting point of our discussion is a fixed initial shape *Ω*. Then, we consider the mapping:

56 $$ V\mapsto g(V) := \mathcal{J}((\text{Id}+V)(\varOmega)) $$

defined on a suitable space of vector fields *Θ* ⊂ *C*^0,1^(*D*)^*d*^. Since the mapping *g* is defined on an open subset *Θ* of the Banach space *C*^0,1^(*D*)^*d*^, we can employ standard algorithms to minimise *g* over *Θ*. The only constraint we must impose is that Id + *V* remains invertible, which can be difficult in practice. In view of $g(V+t W) = \mathcal {J}((\text {Id}+V+tW)(\varOmega )) = \mathcal {J}((\text {Id} + t W\circ (\text {Id}+V)^{-1} )((\text {Id}+V)(\varOmega )))$ for *V*,*W* ∈*Θ* and *t* small, we find by differentiating with respect to *t* at *t* = 0, that:

57 $$ \partial g(V)(W) = D \mathcal{J}((\text{Id}+V)(\varOmega))(W\circ (\text{Id} + V)^{-1}) $$

for *V*,*W* ∈*Θ* and Id + *V* invertible.

### Gradient computation

The gradient of *∂g*(*V* ) in a Hilbert space *H* ⊂ *C*^0,1^(*D*)^*d*^ is defined by:

58 $$  \partial g(V)(W) = (\nabla^H g(V),W)_H \quad \text{ for all }W\in H. $$

Typical choices for *H* are:

59 $$ \begin{array}{@{}rcl@{}} H={H^{1}_{0}}(\mathsf{D})^{d},  (W,V)_{H}&:=& {\int}_{\mathsf{D}} \partial W:\partial V+V\cdot W \text{dx}, \end{array} $$

60 $$ \begin{array}{@{}rcl@{}} H={H^{1}_{0}}(\mathsf{D})^{d},  (W,V)_{H}&:=& {\int}_{\mathsf{D}} \varepsilon(W):\varepsilon(V)+V\cdot W \text{dx},\\ \end{array} $$

61 $$ \begin{array}{@{}rcl@{}} H={H^{1}_{0}}(\mathsf{D})^{d},  (W,V)_{H}&:=& {\int}_{\mathsf{D}} \varepsilon(W):\varepsilon(V)+ V\cdot W \\ && \quad + \gamma_{CR} \mathcal{B} V\cdot \mathcal{B} W \text{dx}, \end{array} $$

where $\varepsilon (V):= \frac 12(\partial V+\partial V^{\top })$, *γ*_*CR*_ > 0 and

62 $$ \mathcal{B} := \begin{pmatrix} -\partial_x & \partial_y \\ \partial_y & \partial_x \end{pmatrix}. $$

The last choice, which is restricted to the spatial dimension *d* = 2, corresponds to a penalised Cauchy-Riemann gradient and results in a gradient which is approximately conformal and hence preserves good mesh quality. We refer to Iglesias et al. (2018) for a detailed description. We also refer to de Gournay (2006) and Burger (2002) and Allaire et al. (2021) for the use of different inner products.

### Basic algorithm

Let *Ω* be an initial shape and let *H* ⊂ *C*^0,1^(*D*)^*d*^ be a Hilbert space. Then, a basic shape optimisation algorithm reads as follows. 
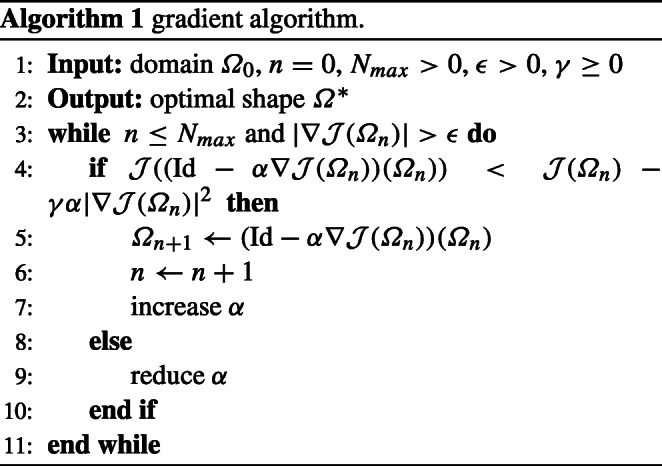


We present and explain the numerical realisation of Algorithm 1 in NGSolve for the case of a PDE-constrained shape optimisation problem in two space dimensions. The simpler case of an unconstrained shape optimisation problem or the case of three space dimensions can be realised by small modifications of the presented code.

First of all, we mention that we realise shape modifications in NGSolve by means of deformation vector fields without actually modifying the coordinates of the underlying finite element grid. Recall the vector-valued finite element space VEC over a given mesh as introduced in code line 44. We define a vector-valued GridFunction with the name gfset which will represent the current shape. We initialise it with some vector-valued coefficient function $V(x_{1}, x_{2}) = ({x_{1}^{2}} x_{2}, {x_{2}^{2}} x_{1})^{\top }$ and obtain the deformed shape (Id + *V* )(*Ω*) by the command mesh.SetDeformation(gfset): 
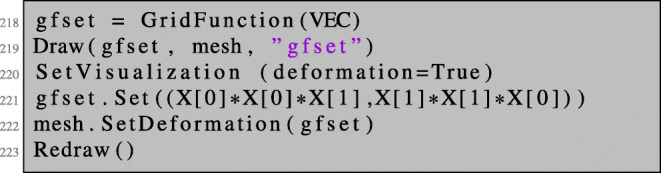


Any operation involving the mesh such as integration or assembling of matrices is now carried out for the deformed configuration. To be more precise, a change of variables is performed internally by accounting for the corresponding Jacobi determinant and transforming the derivatives accordingly with the Jacobian of the deformation. Therefore, all resulting coefficient vectors (which are stored in GridFunction s) correspond to the shape functions in reference configuration. The deformation can be unset by the command mesh.UnsetDeformation(). Integrating the constant function over the mesh in the perturbed and unperturbed settings, 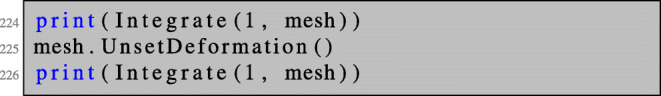


gives the output


1.79245290468626270.7854072970684544

respectively.

In the course of the optimisation algorithm, the state equation as well as the adjoint equation has to be solved for every new shape. We define the following function, which computes the state and adjoint state for a linear PDE constraint: 
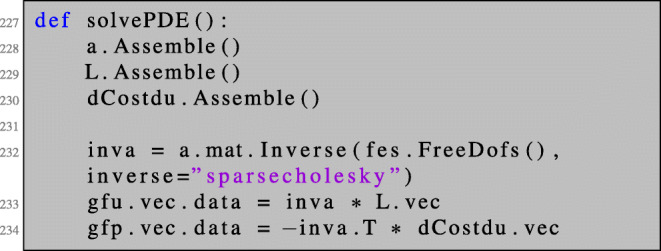


The shape derivative dJOmega for some problem at hand can be defined as illustrated in Sections 4.1 and Section 5. Finally, we need to define the shape gradient, which is the solution to a boundary value problem of the form (58). We choose the bilinear form defined in (61) with *γ*_*CR*_ = 10: 
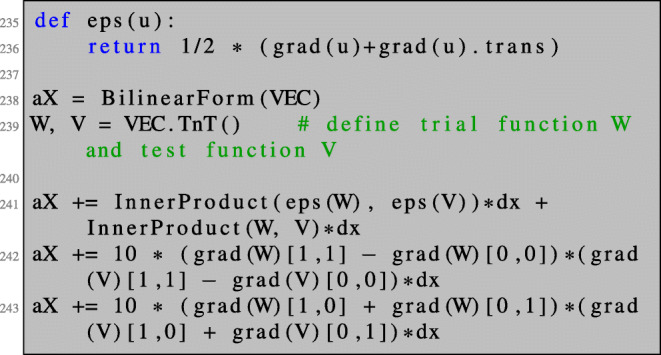


Now, we can run Algorithm 1 for problem (28): 
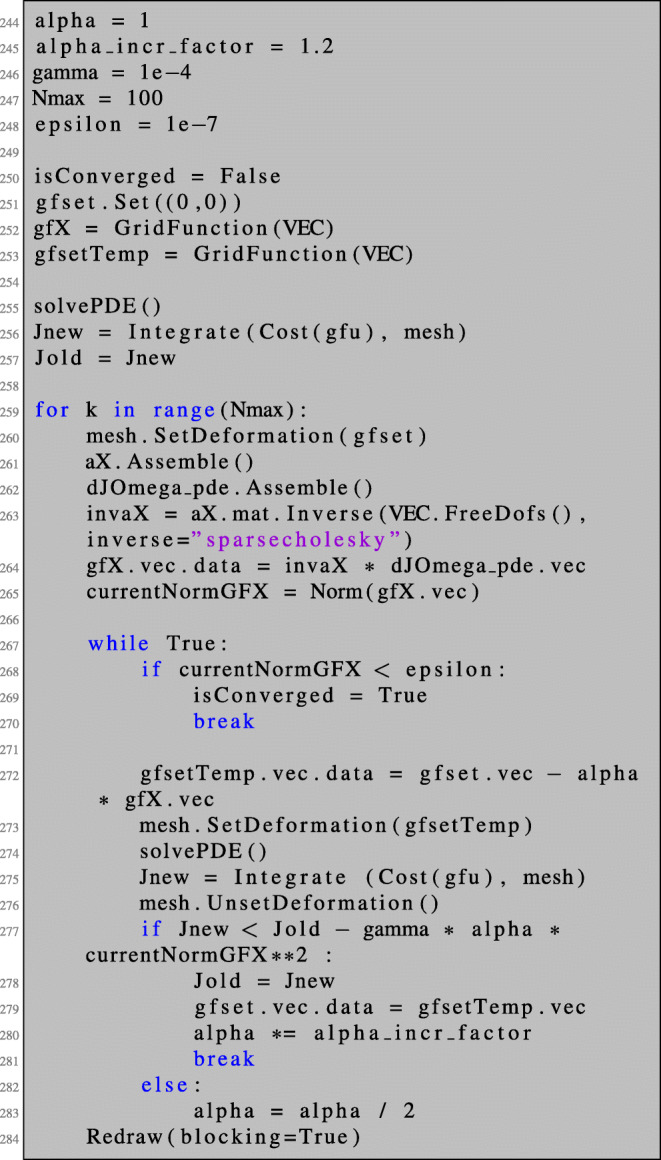


#### Mesh movement and mesh optimisation

As an alternative to realizing the deformations via mesh.SetDeformation(...), where the underlying mesh is not modified, one could also just move every mesh node in the direction of the given descent vector field by changing its coordinates. This can be realised by invoking the following method: 
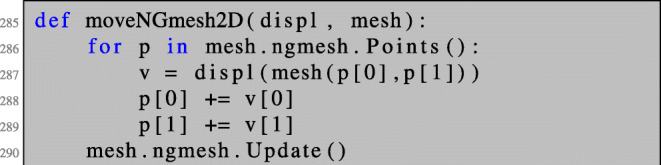


Here, the displacement vector field displ, which is of type GridFunction, is evaluated for each mesh node and, subsequently, the mesh nodes are updated. At the end of the procedure, the mesh structure needs to be updated; see line 290. Note that GridFunction s can only be evaluated at points inside the mesh (but not necessarily vertices of the mesh). Therefore, in order to evaluate displ at the point given by the coordinates p[0], p[1], we need to pass mesh(p[0],p[1]) in line 287.

**Fig. 3:**
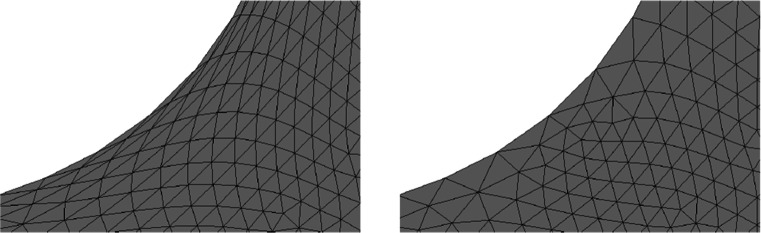
Before and after mesh optimisation by mesh. ngmesh.OptimizeMesh2d()

One advantage of this strategy is that an ill-shaped mesh can easily be repaired by a call of the method mesh.ngmesh.OptimizeMesh2d() followed by mesh.ngmesh.Update(). Figure 3 shows an ill-shaped mesh and the result of a call of mesh.ngmesh.OptimizeMesh2d().

### Newton’s method for unconstrained problems

The particular choice $H={H^{1}_{0}}(\mathsf {D})^{d}$ and

63 $$  (V,W)_H := D^{2}\mathcal{J}(\varOmega)(V)(W), $$

for a given shape function $\mathcal {J}$ leads to Newton’s method. We refer to Novruzi and Roche (2000), Allaire et al. (2016), Paganini and Sturm (2019), and Eppler et al. (2007) where shape Newton methods were used previously and to (Hinze et al. 2009, Chapter 2) and (Ito and Kunisch 2008, Chapter 5) for Newton’s method in an optimal control setting. This bilinear form is only positive semi-definite on ${H^{1}_{0}}(\mathsf {D})^{d}$ since $ D^{2}\mathcal {J}(\varOmega )(V)(W)=0$ for *V*,*W* with *V* = *W* = 0 on *∂Ω*. Moreover, from the structure theorem for second shape derivatives proved in Novruzi and Pierre (2002), we know that at a stationary point *Ω*, that is, $D\mathcal {J}(\varOmega )(V)=0$ for all *V* ∈ *C*^0,1^(*D*)^*d*^, we have

64 $$ D^{2}\mathcal{J}(\varOmega)(V)(W) = \ell_\varOmega(V\cdot n,W\cdot n), $$

where *ℓ*_*Ω*_ : *C*^0^(*∂Ω*) × *C*^0^(*∂Ω*) →**R** is a bilinear function. Hence, we also have $D^{2}\mathcal {J}(\varOmega )(V)(W)=0$ for all *V*,*W* such that *V* ⋅ *n* = *W* ⋅ *n* = 0. As a result, the gradient

65 $$  (\nabla \mathcal{J}(\varOmega),V)_H = D\mathcal{J}(\varOmega)(V) \quad\text{ for all }V\in H^1_0(\mathsf{D})^{d} $$

according to (63) is not uniquely determined. To get around this difficulty, the shape Hessian is often regularised by an *H*^1^ term, i.e. (63) is replaced by

66 $$  D^{2}\mathcal{J}(\varOmega)(V)(W) + \delta {\int}_\varOmega \partial V : \partial W + V \cdot W  \text{dx}, $$

see, e.g. Schmidt (2018), which, however, impairs the convergence speed of Newton’s method.

#### Alternative regularisation strategy

Here, we propose the following strategy: We regularise the shape Hessian only on the boundary *∂Ω* and only in tangential direction, i.e. we choose

67 $$  (V,W)_H := D^{2}\mathcal{J}(\varOmega)(V)(W) + \delta {\int}_{\partial \varOmega} (V\cdot \tau)(W \cdot \tau) $$

with a regularisation parameter *δ*. To exclude the part of the kernel corresponding to interior deformations, we solve the (regularised) Newton (65) only on the boundary *∂Ω*. This is realised by setting Dirichlet boundary conditions for all degrees of freedom except those on the boundary. 
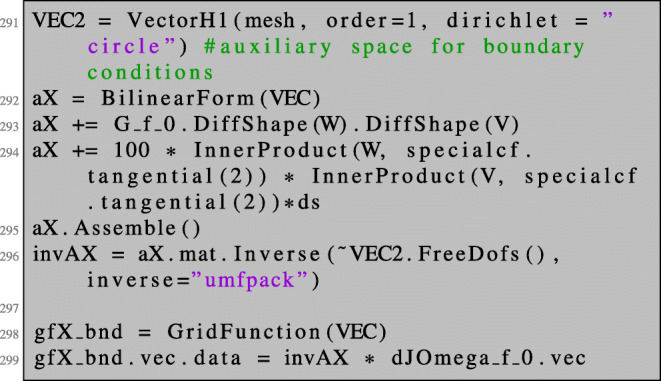


As a result, we get a shape gradient $\tilde {\nabla \mathcal {J}(\varOmega )}$ which is nonzero only on the boundary. We extend this vector field to the interior by solving an additional boundary value problem (of linearised elasticity type), where we use the deformation given by $\tilde {\nabla \mathcal {J}(\varOmega )}$ as Dirichlet boundary conditions. 
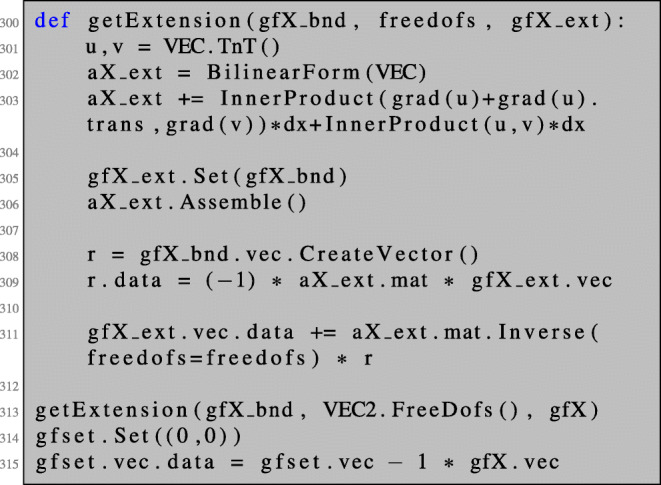


The Newton algorithm reads as follows. 
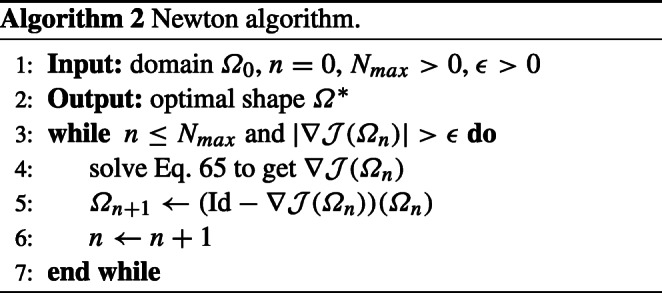


### Newton’s method for PDE-constrained problems

We consider the PDE-constrained model problem of Section 4.1 which is subject to the Poisson equation. The unregularised Newton system reads:

68 $$  D^{2}\mathcal{J}(\varOmega)(V)(W) = - D\mathcal{J}(\varOmega)(V) \quad \text{ for all } V\in H^1_0(\varOmega). $$

In Section 4.3, we discussed how the second-order shape derivative can be evaluated along a fixed given direction. In this section, we want to assemble the whole shape Hessian and eventually solve a regularised version of (68). Recalling that $D\mathcal {J}(\varOmega )(V) = \partial _{s} G_{V,0}(x)$ with *x* = (0,0,*u*,*p*), we see that (47) and (43) lead to:

69 $$  \mathcal H \begin{pmatrix} \tilde V \\ \partial_s u^0 \\ \partial_s p^0 \end{pmatrix} = - \begin{pmatrix} \partial_t G_{V,W}(x) \\ 0\\ 0 \end{pmatrix}. $$

with

70 $$ \mathcal H(x) = \begin{pmatrix} \partial_s\partial_t G_{V,W}(x) & \partial_u \partial_t G_{V,W}(x) & \partial_p \partial_t G_{V,W}(x) \\ \partial_s \partial_u G_{V,W}(x)   & \partial_u^2G_{V,W}(x) & \partial_p\partial_u G_{V,W}(x) \\ \partial_s \partial_p G_{V,W}(x)  & \partial_u \partial_p G_{V,W}(x) & 0 \end{pmatrix}. $$

The component $\tilde V$ then represents the direction which we use for the shape Newton optimisation step. The matrix in (69) can be realised in NGSolve by using a combined finite element space X3 consisting of three components as follows: 
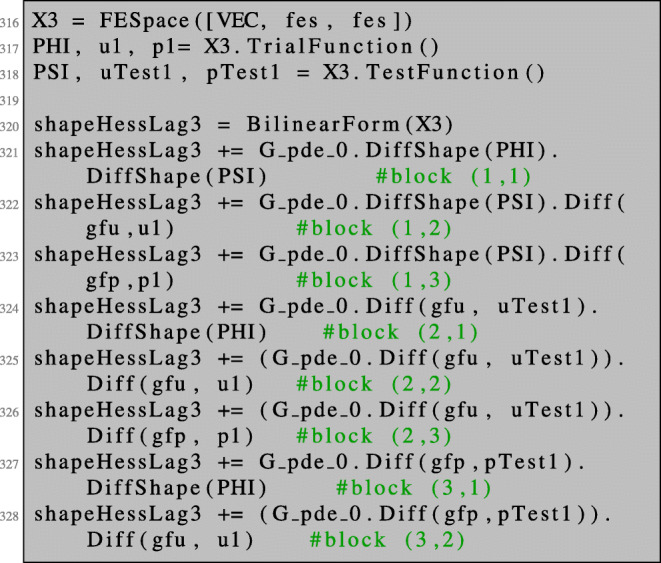


The right-hand side of (69) can be defined as follows: 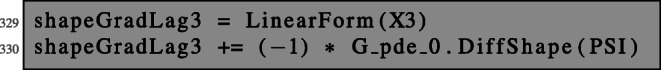


Recall that the system (65) has a nontrivial kernel as discussed in Section 6.3. This problem can be circumvented by proceeding like in the unconstrained case. We add a regularisation only on the boundary, 
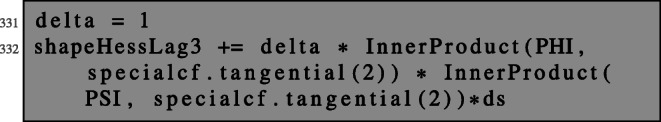


and exclude the interior degrees of freedom in the first row and column of the 3×3 block system. This can be realised by setting Dirichlet boundary conditions for the interior degrees of freedom, i.e. by dealing with the free degrees of freedom, 
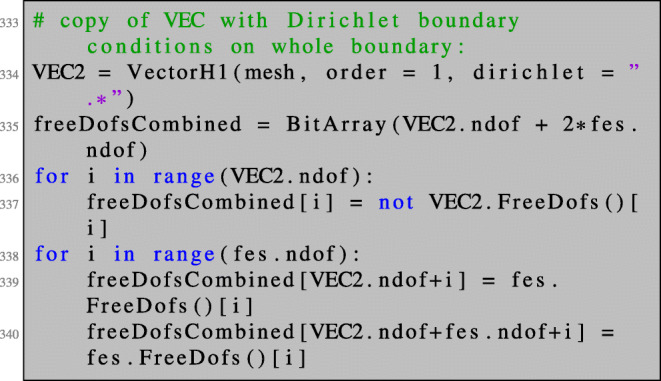


and solving the regularised system using these free dofs: 
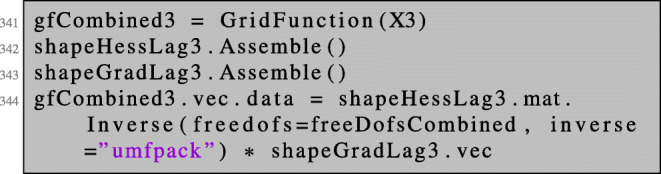


The Newton direction is then given as the first of the three components of the obtained solution. 
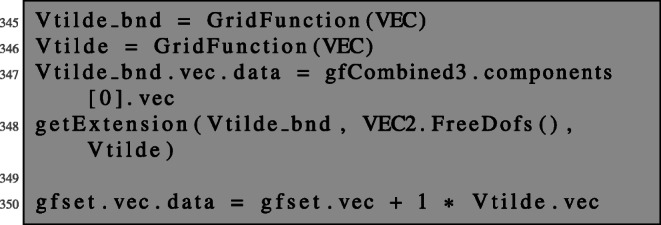


## Numerical experiments

In this section, we first verify the computed shape derivatives by performing a Taylor test, and then apply the automated shape differentiation and the numerical algorithms introduced in the preceding sections in numerical examples.

### Code verification

We verify the expressions that we obtained in a semi-automatic or fully automatic way for the first- and second-order shape derivatives by looking at the Taylor expansions of the perturbed shape functionals. We illustrate our findings in two examples in **R**^2^. On the one hand, we consider a shape function as introduced in (6) with an additional boundary integral as in (13), henceforth denoted by $\mathcal {J}_{1}$; on the other hand, we consider the PDE-constrained shape optimisation problem defined by (28), the reduced form of which will be denoted by $\mathcal {J}_{2}(\varOmega )$. More precisely, we consider:

71 $$ \begin{array}{@{}rcl@{}} \mathcal{J}_{1}(\varOmega) &=& {\int}_{\varOmega} f(x)  \text{dx} + {\int}_{\partial \varOmega} f(x)  \text{ds}, \end{array} $$

72 $$ \begin{array}{@{}rcl@{}} \mathcal{J}_{2}(\varOmega) &=& {\int}_{\varOmega} |u_{\varOmega}-u_{d}|^{2}  \text{dx}  \text{ where } u_{\varOmega} \text{ solves (28b). } \end{array} $$

In the case of $\mathcal {J}_{1}$, we used the function:

$$f(x_{1}, x_{2}) = \left( 0.5+\sqrt{{x_{1}^{2}} +{x_{2}^{2}}}\right)^{2} \left( 0.5-\sqrt{{x_{1}^{2}}+{x_{2}^{2}}} \right)^{2}$$

and for $\mathcal {J}_{2}$, we used *u*_*d*_(*x*_1_,*x*_2_) = *x*_1_(1 − *x*_1_)*x*_2_(1 − *x*_2_) and *f*(*x*_1_,*x*_2_) = 2*x*_2_(1 − *x*_2_) + 2*x*_1_(1 − *x*_1_) for the function *f* in the PDE constraint (28b).

For the test of the first-order shape derivatives $D \mathcal {J}_{i}(\varOmega )(V)$, we choose a fixed shape *Ω* and a vector field *V* ∈ *C*^0,1^(**R**^2^)^2^ and observe the quantity:

73 $$ \delta_1(\mathcal{J}_i, t) := \left\lvert \mathcal{J}_i((\text{Id} + t V)(\varOmega) ) - \mathcal{J}_i(\varOmega) - t  D\mathcal{J}_i(\varOmega)(V) \right\rvert, $$

for *t* ↘ 0. Likewise, for the second-order shape derivative, we consider the remainder:

$$ \begin{array}{@{}rcl@{}} \delta_{2}(\mathcal{J}_{i}, t) := \left\lvert \vphantom{\frac{1}{2}}\right. \mathcal{J}_{i}((\text{Id} + t V )(\varOmega) ) &- \mathcal{J}_{i}(\varOmega) - t  D\mathcal{J}_{i}(\varOmega)(V) \\ &- \frac{1}{2} t^{2} D^{2} \mathcal{J}_{i}(\varOmega)(V)(V) \left. \vphantom{\frac{1}{2}} \right\rvert \end{array} $$

as *t* ↘ 0. By the definition of first- and second-order shape derivatives, it must hold that

74 $$ \delta_1(\mathcal{J}_i, t) = \mathcal O(t^2) \quad \text{ and } \quad \delta_2(\mathcal{J}_i, t) = \mathcal O(t^3) \quad \text{ as } t \searrow 0. $$

**Fig. 4:**
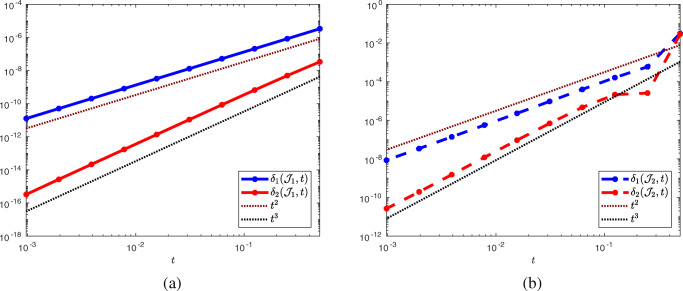
Taylor test for functions $\mathcal {J}_{1}$ and $\mathcal {J}_{2}$

This behavior can be observed in Fig. 4a for $\mathcal {J}_{1}$ and in Fig. 4b for $\mathcal {J}_{2}$, where we used $V(x_{1}, x_{2}) = ({x_{1}^{2}} x_{2} e^{x_{2}}, {x_{2}^{2}} x_{1} e^{x_{1}})$ in both cases.

The experiments for shape function $\mathcal {J}_{1}$ were conducted on a mesh consisting of 13,662 vertices, 26,946 elements, and with polynomial order 2 (resulting in 54,269 degrees of freedom), and the experiment for $\mathcal {J}_{2}$ with 95,556 vertices and 190,062 elements and polynomial degree 1 (95,556 degrees of freedom). We conducted these experiments for a number of different problems with different vector fields *V*, in particular with different PDE constraints and boundary conditions, and obtained similar results in all instances provided a sufficiently fine mesh was used.

### A first shape optimisation problem

In this section, we revisit problem (6) introduced in Section 3, i.e. the problem of finding a shape *Ω* such that the cost function $\mathcal {J}(\varOmega ) = {\int \limits }_{\varOmega } f(x) \text {dx}$ is minimised.

#### First-order methods

**Fig. 5:**
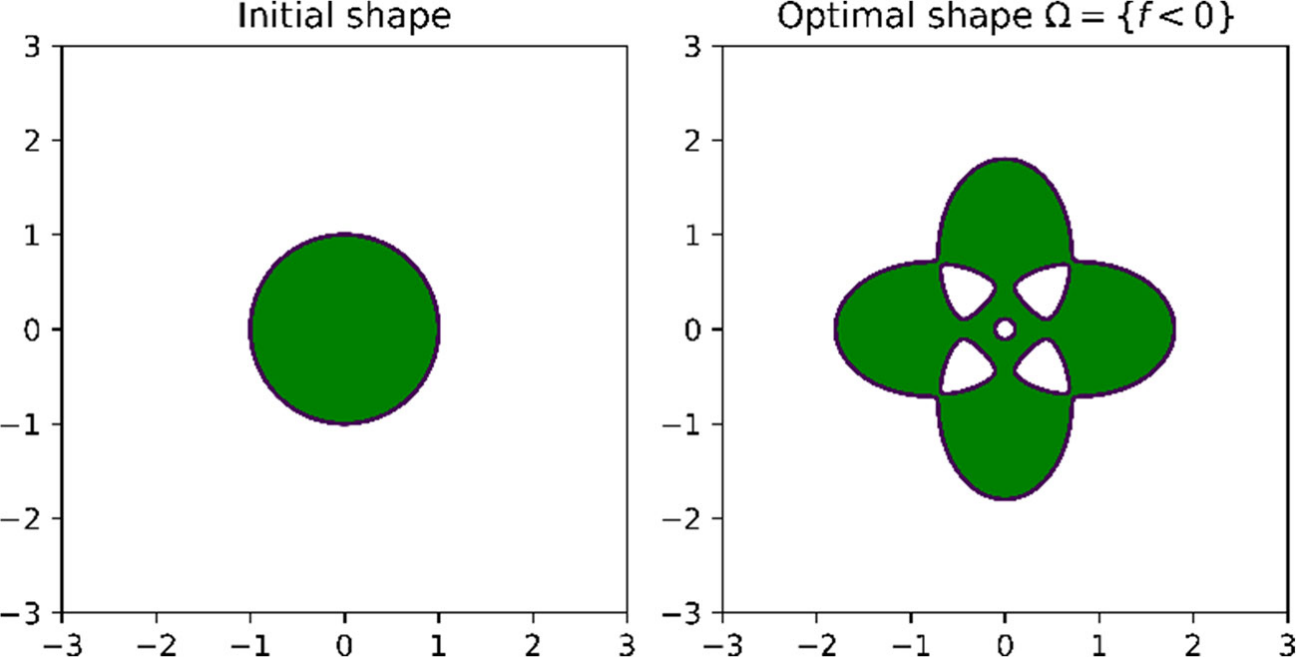
Initial domain *Ω*_0_ and optimal domain *Ω*^∗^ for problem (6) with *f* chosen according to (75)

We illustrate our first-order methods in a problem which was also considered in Iglesias et al. (2018) and reproduce the results obtained there. We choose the function:

75 $$ \begin{array}{@{}rcl@{}} f(x_{1},& x_{2}) = \left( \sqrt{(x_{1} - a)^{2} + b {x_{2}^{2}}} - 1\right) \left( \sqrt{(x_{1} + a)^{2} + b {x_{2}^{2}}} - 1\right) \\ & \cdot \left( \sqrt{b {x_{1}^{2}} + (x_{2} - a)^{2}} - 1\right) \left( \sqrt{b {x_{1}^{2}} + (x_{2} + a)^{2}} - 1\right) - \epsilon \end{array} $$

with $a=\frac {4}{5}$, *b* = 2 and *𝜖* = 0.001. Recall that the optimal shape is given by $\{(x_{1},x_{2}) \in \mathbf {R}^{2}: f(x_{1}, x_{2})<0 \}$ which is depicted in Fig. 5 (right). We start our optimisation algorithm with the unit disk, *Ω*^0^ = *B*_1_(0) as an initial design. Note that the optimal design cannot be reached by means of shape optimisation using boundary perturbations. However, we expect the outer curve of the optimal shape to be reached very closely.

**Fig. 6:**
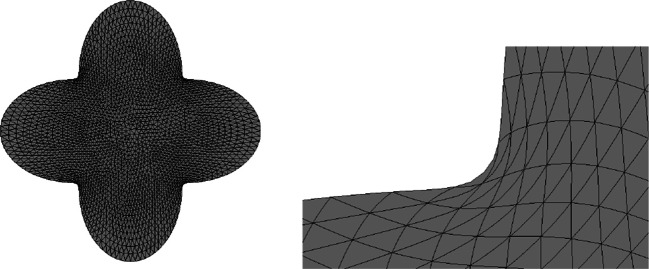
Results of problem (6) with *f* as in (75) and the shape gradient associated to the *H*^1^ inner product (59)

**Fig. 7:**
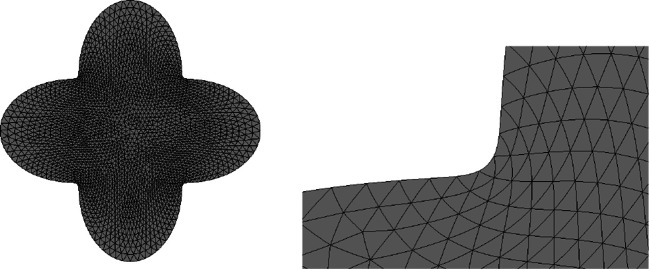
Results of problem (6) with *f* as in (75) and the shape gradient associated to the elasticity bilinear form (60)

**Fig. 8:**
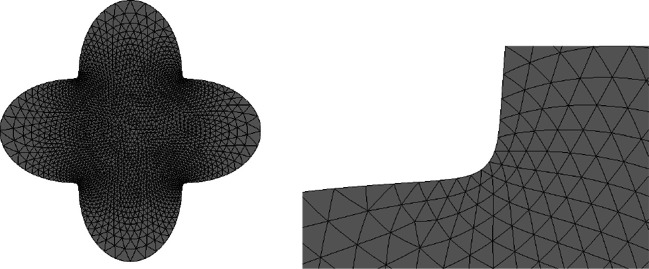
Results of problem (6) with *f* as in (75) and the shape gradient associated to the elasticity bilinear form with Cauchy-Riemann term (61)

We apply Algorithm 1 with the shape gradient $\nabla \mathcal {J}$ associated to the *H*^1^ inner product (59), to the bilinear form of linearised elasticity (60) and including the additional Cauchy-Riemann term (61). We chose the algorithmic parameters *γ* = 1*e* − 4, *𝜖* = 1*e* − 7, a mesh consisting of 2522 vertices and 4886 elements and a globally continuous vector-valued finite element space VEC of order 3. The results can be seen in Figs. 6, 7 and 8, respectively.

#### Second-order method

**Fig. 9:**
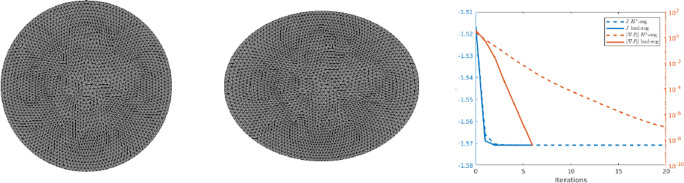
Numerical results for problem (67) with *f* as in (76) using second-order method. Left: Initial design. Centre: Optimised design after six iterations using (65)/(67). Right: Objective value $\mathcal {J}$ and norm of shape gradient $\| \nabla \mathcal {J}(\varOmega )\|$ in the course of second-order optimisation using (66) with *δ* = 0.5 and (67) with *δ* = 100

Since Newton’s method converges quadratically only in a neighborhood of the optimal solution, we choose a simpler optimal design here. We choose:

76 $$  f(x_1, x_2) = \frac{x^2}{a^2} + \frac{y^2}{b^2} - 1 $$

which yields an ellipse with the lengths of the two semi-axes *a* and *b*. We choose *a* = 1.3 and *b* = 1/*a* and again start the optimisation with the unit disk as an initial shape. Figure 9 shows the initial and optimised design after only six iterations of Algorithm 2 with (⋅,⋅)_*H*_ chosen as in (67) with *δ* = 100. A comparison of the convergence histories between the choice (67) with *δ* = 100 and (66) with *δ* = 0.5 is shown in the right picture of Fig. 9. In both cases, we tested a range of different values for *δ* and compared the convergence histories for the values which yielded the fastest convergence. The experiments were conducted on a finite element mesh consisting of 2522 nodes and 4886 triangular elements with a finite element space VEC of order 3, with the algorithmic parameter *𝜖* = 10^− 7^.

### Shape optimisation subject to the Poisson equation

In this section, we revisit the model problem introduced in Section 4.1 with *f*(*x*_1_,*x*_2_) = 2*x*_2_(1 − *x*_2_) + 2*x*_1_(1 − *x*_1_) and *u*_*d*_(*x*_1_,*x*_2_) = *x*_1_(1 − *x*_1_)*x*_2_(1 − *x*_2_). Note that the data is chosen in such a way that, for *Ω*^∗^ = (0,1)^2^ it holds $\mathcal {J}(\varOmega ^{*}) = 0$ and thus *Ω*^∗^ is a global minimiser of $\mathcal {J}$. We show results obtained by first- and second-order shape optimisation methods exploiting automated differentiation.

**Fig. 10:**
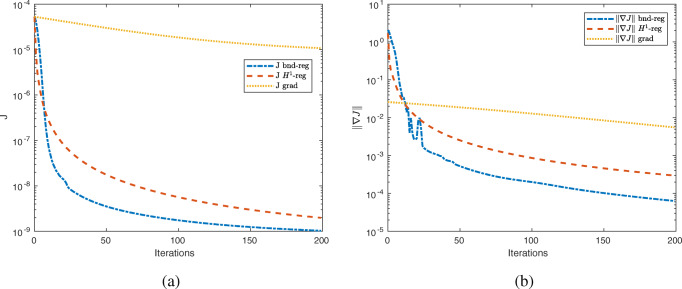
Convergence behaviour for shape optimisation problem (28) with proposed regularisation strategies (67) and (66) as well as first-order method with constant step size *α* = 1. **a** Behaviour of objective function $\mathcal {J}$. **b** Behaviour of norm of shape gradient $\| \nabla \mathcal {J}(\varOmega )\|$

We ran the optimisation algorithm in three versions. On the one hand, we applied a first-order method with constant step size *α* = 1. On the other hand, we applied two second-order methods with the two different regularisation strategies for the shape Hessian in (65) introduced in (66) and (67). We chose the regularisation parameters *δ* empirically such that the method performs as well as possible. In the case of (66), we chose *δ* = 0.001 and in the case of (67) *δ* = 1. The experiments were conducted on a finite element mesh consisting of 4886 elements with 2522 vertices and polynomial degree 1. In Fig. 10, we can observe the decrease of the objective function as well as of the norm of the shape gradient over 200 iterations for these three algorithmic settings.

**Fig. 11:**
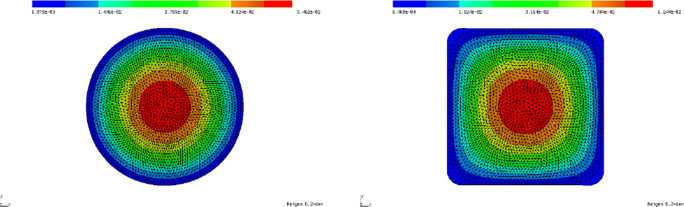
Shape optimisation for problem (28). Left: Initial design. Right: Improved design after 200 iterations of second-order algorithm with regularisation as proposed in (67). Objective value was reduced from 5.297 ⋅ 10^− 5^ to 1.0317 ⋅ 10^− 9^. Colour shows solution of constraining PDE (28b)

Figure 11 shows the initial design as well as the design after 200 iterations of the second-order method with regularisation strategy (67). Note that the improved design is very close to *Ω*^∗^ = (0,1)^2^, which is a global solution. The initial design was chosen as the disk of radius $\frac {1}{2}$ centred at the point $\left (\frac {1}{2}, \frac {1}{2}\right )^{\top }$. The objective value was reduced from 5.297 ⋅ 10^− 5^ to 1.0317 ⋅ 10^− 9^.

### Nonlinear elasticity

**Fig. 12:**
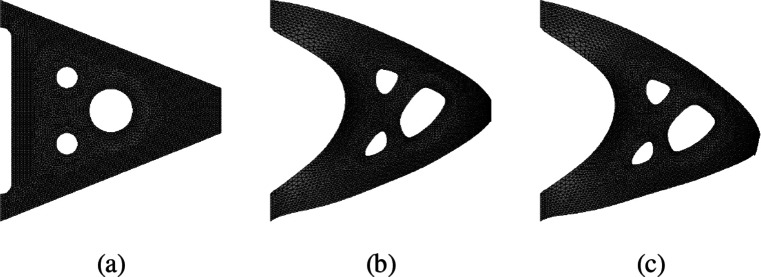
Initial and optimised geometry of cantilever under vertical force on right-hand side using St. Venant–Kirchhoff model in nonlinear elasticity. **a** Initial geometry. **b** Optimised geometry (reference configuration). **c** Optimised geometry (deformed configuration)

Here, we illustrate the applicability of the automated shape differentiation and optimisation in the more realistic and more complicated setting of nonlinear elasticity in two space dimensions using a Saint Venant–Kirchhoff material with Young’s modulus *E* = 1000 and Poisson ratio *ν* = 0.3. We consider a two-dimensional cantilever which is clamped on the upper and lower left parts of the boundary, ${{\Gamma }_{l}^{1}} = \{0\} \times (0.88, 1)$ and ${{\Gamma }_{l}^{2}} = \{0\} \times (0, 0.12)$, respectively, and is subject to a surface force *g*_*N*_ = (0,− 100)^⊤^ on Γ_*r*_ = {1}× (0.45,0.55). The initial geometry with 3 holes is depicted in Fig. 12a. Let ${\Gamma }_{l} := {{\Gamma }_{l}^{1}} \cup {{\Gamma }_{l}^{2}}$ and $H_{{\Gamma }_{l}}^{1}(\varOmega )^{2}$ the subspace of *H*^1^(*Ω*)^2^ with vanishing trace on Γ_*l*_. The displacement $u\in H_{{\Gamma }_{l}}^{1}(\varOmega )^{2}$ under the surface force *g*_*N*_ is given as the solution to the boundary value problem:

77 $$  {\int}_\varOmega S(u) : \nabla v  \text{dx} = {\int}_{{\Gamma}_r} g_N \cdot v  \text{ds} $$

for all $v \in H_{{\Gamma }_{l}}^{1}(\varOmega )^{2}$. Here, *S*(*u*) denotes the Saint Venant–Kirchhoff stress tensor:

78 $$ S(u) = (I_2+\nabla u) \left[\lambda \text{Tr}\left( \frac{1}{2} (C(u) - I_2) \right)I_2 + \mu(C(u) - I_2) \right], $$

where *C*(*u*) = (*I*_2_ + ∇*u*)^⊤^(*I*_2_ + ∇*u*) and *I*_2_ is the identity matrix (see also (Allaire et al. 2004, Sec. 8)), and *λ* and *μ* denote the Lamé constants:

79 $$ \lambda = \frac{E \nu }{(1+\nu)(1-2\nu)},\qquad \mu = \frac{E}{2 (1+\nu)}. $$

We minimise the functional

80 $$ J(\varOmega, u) = {\int}_\varOmega S(u) : \nabla u  \text{dx} +\alpha {\int}_\varOmega 1  \text{dx} $$

with *α* = 2.5 subject to (77) which amounts to maximising the structure’s stiffness while bounding the allowed amount of material used.

We remark that the well-posedness of (77) is not clear (see also the discussion in (Allaire et al. 2004, Sec. 8)). Nevertheless, application of the automated shape differentiation and optimisation yields a significant improvement of the initial design. The highly nonlinear PDE constraint (77) is solved by Newton’s method. In order to have good starting values, a load stepping strategy is employed, i.e. the load on the right-hand side is gradually increased, the PDE is solved and the solution is used as an initial guess for the next load step. This is repeated until the full load is applied. With these ingredients at hand, Algorithm 1 (i.e. code lines 244–284) can be run. We chose the algorithmic parameters alpha = 0.1 (as an initial value), alpha_incr_factor = 1 (i.e. no increase), gamma = 1e-4 and epsilon = 1e-7. Moreover, we used (59) with an additional Cauchy-Riemann term as in (61) with weight *γ*_*CR*_ = 10. The objective value was reduced from 3.125 to 2.635 (volume term from 1.290 to 1.096) in 15 iterations of Algorithm 1. The results were obtained on a mesh consisting of 10,614 elements and 5540 vertices using piecewise linear, globally continuous finite elements.

### Helmholtz equation

**Fig. 13:**
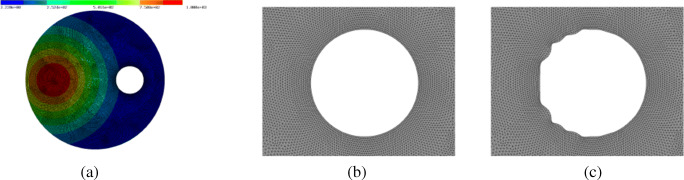
**a** Geometry with the right-hand side *f*. **b** Initial shape of scatterer (zoom of geometry in **a**). **c** Optimised shape of scatterer (zoom)

In this section, we consider the problem of finding the optimal shape of a scattering object. More precisely, we consider the minimisation of the functional:

81 $$  {\int}_{{\Gamma}_r} u \bar{u}  \text{ds} $$

subject to the Helmholtz equation with impedance boundary conditions on the outer boundary: Find *u* ∈ *H*^1^(*Ω*,**C**) such that

82 $$  {\int}_\varOmega [ \nabla u \cdot \nabla \bar w - \omega^2 u \bar w \big]  \text{dx} - i \omega {\int}_{\Gamma} u \bar w  \text{ds} = {\int}_{\varOmega} f \bar w $$

for all *w* ∈ *H*^1^(*Ω*,**C**). Here, $\bar {w}$ denotes the complex conjugate of a complex-valued function *w*, *ω* denotes the wave number, *i* denotes the complex unit and the function *f* on the right-hand side is chosen as

83 $$ f(x_1, x_2) = 10^3 \cdot e^{-9((x_1-0.2)^2 + (x_2-0.5)^2)} $$

(see Fig. 13a). Furthermore,

$$\varOmega = B((0.5,0.5)^{\top},1) \setminus B((0.75, 0.5)^{\top}, 0.15)$$

denotes the domain of interest, ${\Gamma } = \{(x_{1}, x_{2}): {x_{1}^{2}} + {x_{2}^{2}} =1 \}$ the outer boundary and ${\Gamma }_{r}= \{(x_{1}, x_{2}): {x_{1}^{2}} + {x_{2}^{2}} =1, x_{1} \geq 0 \}$ the right half of the outer boundary. Here, only the inner boundary *∂Ω* ∖Γ is subject to the shape optimisation. Thus, the aim of this model problem is to find a shape of the scattering object such that the waves are reflected away from Γ_*r*_.

**Fig. 14:**
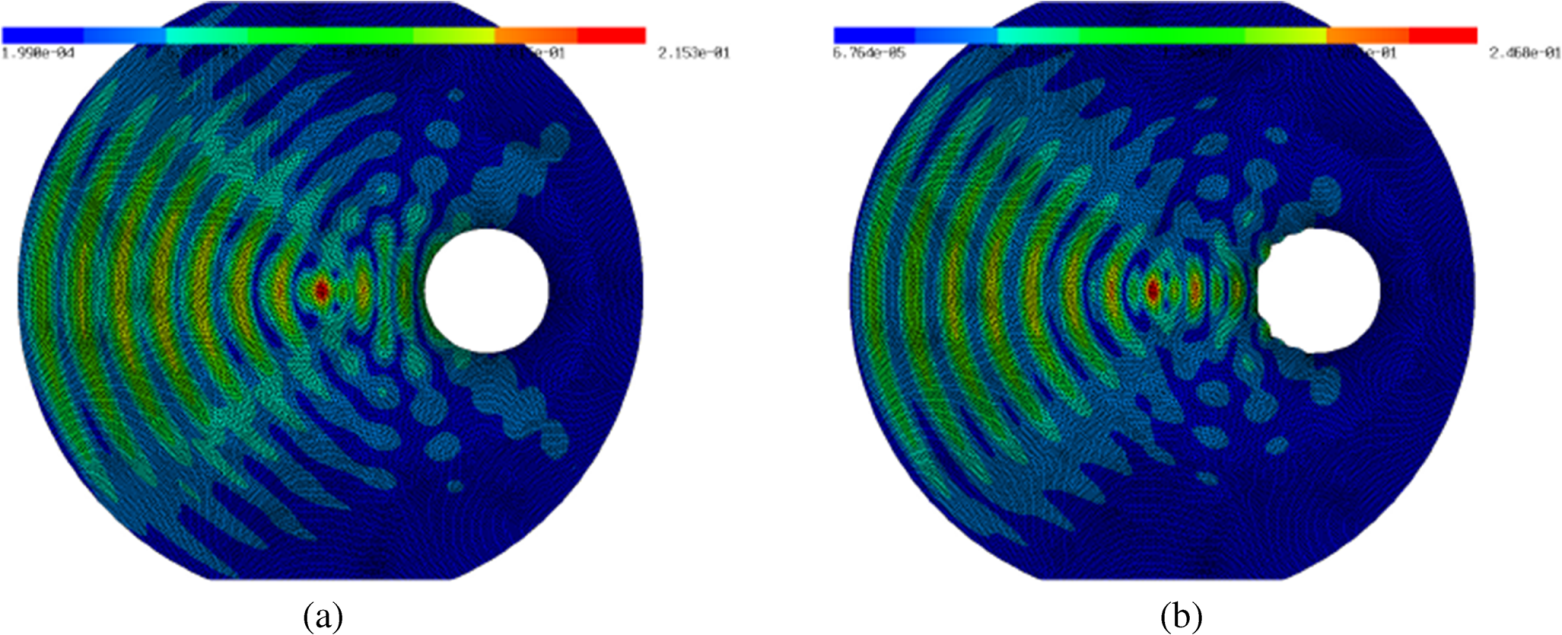
**a** Absolute value of state *u* for initial configuration. **b** Absolute value of state *u* for optimised configuration

Figure 13b and c show the initial and final shape of the scattering object, respectively. Figure 14 shows the norm of the state for the initial configuration (circular shape of scattering object) and for the optimised configuration. The objective value was reduced from 3.44 ⋅ 10^− 3^ to 3.31 ⋅ 10^− 3^. The forward simulations were performed using piecewise linear finite elements on a triangular grid consisting of 34,803 degrees of freedom. The optimisation stopped after 12 iterations.

### Application to an electric machine

**Fig. 15:**
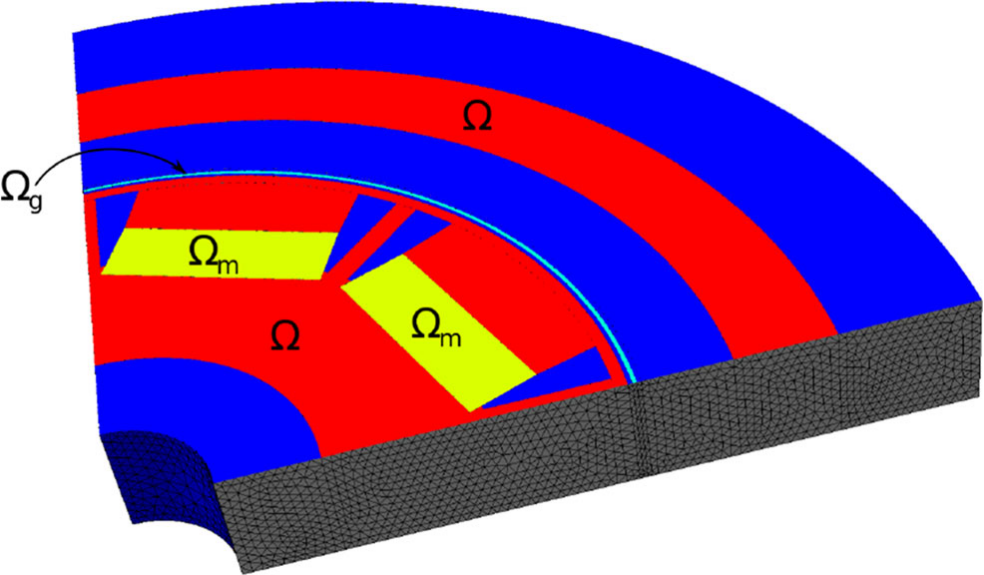
Geometry of electric motor with subdomains in 2D cross section. The ferromagnetic subdomains *Ω* are depicted in red, *Ω*_*m*_ corresponds to the permanent magnets. The rest of the computational domain represents air. Furthermore, *Ω*_*g*_ represents the air gap region that is relevant for the cost function (84)

In this section, we consider the setting of three-dimensional nonlinear magnetostatics in *H*(**curl**,*D*) as it appears in the simulation of electric machines. Let *D* ⊂**R**^3^ denote the computational domain, which consists of ferromagnetic material, air regions and permanent magnets (see Fig. 15). Our aim is to minimise the functional

84 $$  {\int}_{\varOmega_g} | \text{\textbf{curl}} u \cdot n - B_d^n|^2  \text{dx}, $$

where *Ω*_*g*_ denotes the air gap region of the machine, *n* denotes an extension of the normal vector to the interior of *Ω*_*g*_, ${B_{d}^{n}}: \varOmega _{g} \rightarrow \mathbf {R}^{3}$ is a given smooth function and *u* ∈ *H*_0_(**curl**,*D*) is the solution to the boundary value problem

85 $$  {\int}_D \nu_{\varOmega}(|\text{\textbf{curl}} u|) \text{\textbf{curl}} u \cdot \text{\textbf{curl}} w + \delta u \cdot w  \text{dx} = {\int}_{\varOmega_m} M \cdot \text{\textbf{curl}} w  \text{dx} $$

for all *w* ∈ *H*_0_(**curl**,*D*). Here, *Ω* ⊂*D* denotes the union of the ferromagnetic parts of the electric machine, *Ω*_*m*_ denotes the permanent magnets subdomain and

86 $$ \nu_\varOmega = \chi_\varOmega(x) \hat \nu (|\text{\textbf{curl}} u|) + \chi_{\mathsf{D} \setminus \varOmega}(x) \nu_0 $$

denotes the magnetic reluctivity, which is a nonlinear function $\hat \nu $ inside the ferromagnetic regions and equal to a constant *ν*_0_ elsewhere. Furthermore, *δ* > 0 is a small regularisation parameter and $M:\mathsf {D} \rightarrow \mathbf {R}^{3}$ denotes the magnetisation in the permanent magnets. The nonlinear function $\hat \nu $ satisfies a Lipschitz condition and a strong monotonicity condition such that problem (85) is well-posed. The goal of minimising the cost function (84) is to obtain a design which exhibits a smooth rotation pattern. Note that in this particular example we do not consider rotation of the machine, but rather a fixed rotor position, and there are no electric currents present. We refer the reader to (Gangl and Sturm 2019, Sec. 6) for a more detailed description of the problem and to (Gangl et al. 2015) for a 2D version of the same problem.

As mentioned in Section 4, the transformation Φ_*t*_ used in (25) depends on the differential operator. For the **curl**-operator, we have

$$ \begin{array}{@{}rcl@{}} {\Phi}_{t}(u) &= \partial T_{t}^{-\top} (u \circ T_{t}^{-1}) \qquad \qquad \text{ and } \\ (\text{\textbf{curl}} ({\Phi}_{t}(u)))\circ T_{t} &= \frac{1}{\det(\partial T_{t})} \partial T_{t} \text{\textbf{curl}}(u), \end{array} $$

see e.g. (Monk 2003, Section 3.9). Thus, the variational (85) can be defined as follows. 
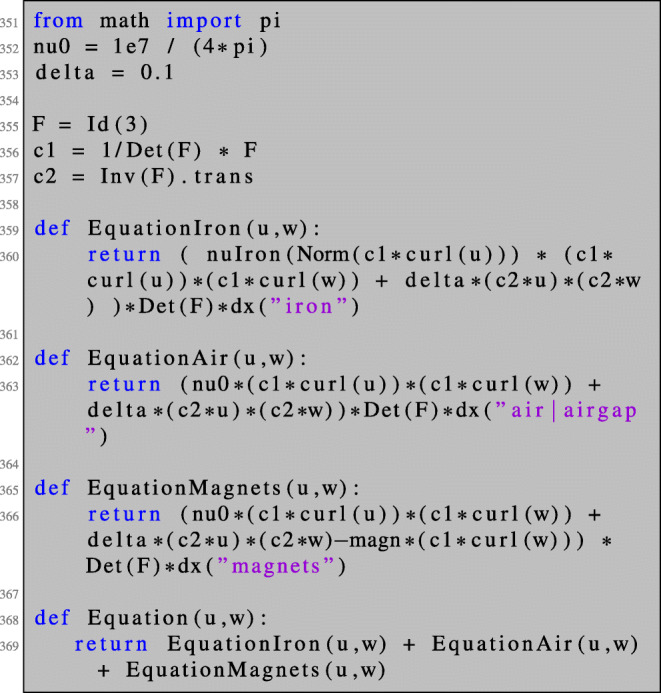


Here, the computational domain consists of a subdomain representing the ferromagnetic part of the machine (‘‘iron'') and a subdomain comprising the permanent magnets (‘‘magnets''); the union of all air subdomains, including the air gap between rotating and fixed part of the machine, is given by ‘‘air|airgap'' (see Fig. 15).

**Fig. 16:**
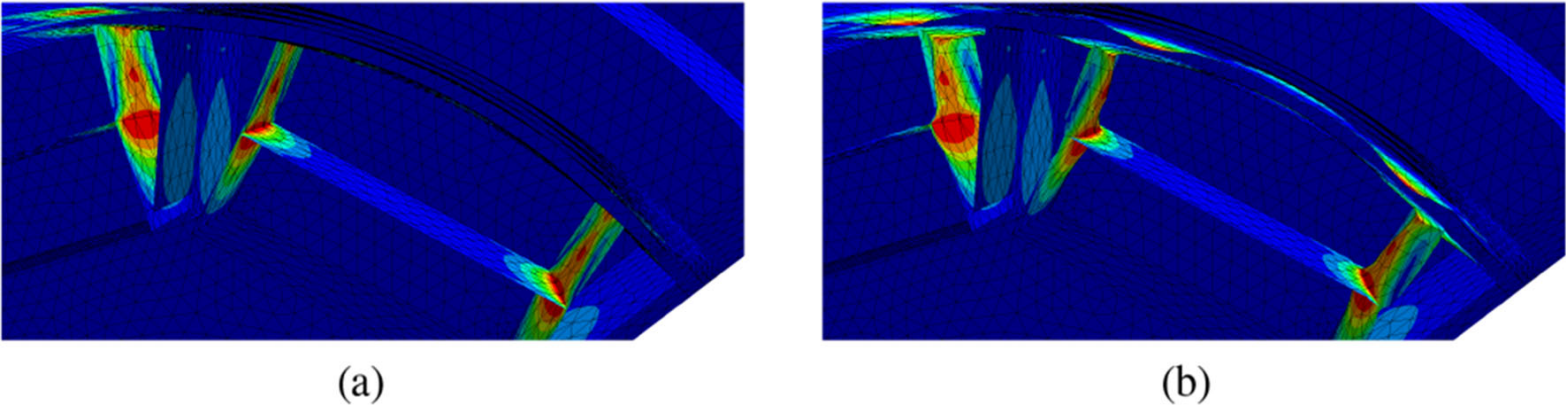
**a** Initial design of electric machine. **b** Optimised design

**Fig. 17:**
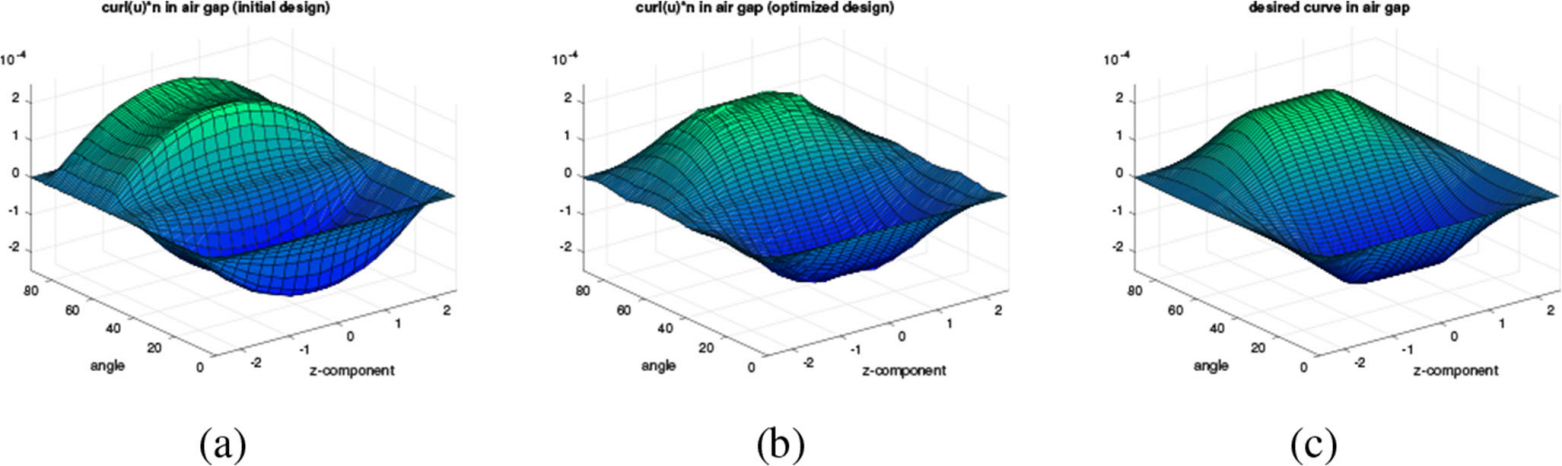
Improvement of **curl***u* ⋅ *n* as a function of *z* and the angle *φ* for a fixed radius *r* inside *Ω*_*g*_ compared to desired curve ${B_{d}^{n}}$. **a** **curl***u* ⋅ *n* for initial configuration. **b** **curl***u* ⋅ *n* for optimised configuration after 10 iterations. **c** Desired curve ${B_{d}^{n}}$ in polar coordinates as function of *z* and the angle *φ* for a fixed radius *r*

Moreover, nuIron denotes the nonlinear reluctivity function $\hat \nu $ and magn contains the magnetisation direction of the permanent magnets. Likewise, the objective function can be defined as follows: 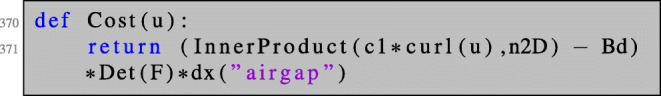


where n2D and Bd represent the extension of the normal vector to the interior of the air gap and the desired curve, respectively. For the definition of all quantities, we refer the reader to Online Resource 1. The shape differentiation as well as the optimisation loop now works in the same way as in the previous examples. Figure 16 shows the initial design of the motor as well as the optimised design obtained after 11 iterations of Algorithm 1 with *γ* = 0. The experiment was conducted using a tetrahedral finite element mesh consisting of 13,440 vertices, 57,806 elements and Nédélec elements of order 2 (resulting in a total of 323,808 degrees of freedom). The objective value was reduced from 2.5944 ⋅ 10^− 8^ to 4.565 ⋅ 10^− 10^ in the course of the first order optimisation algorithm after 11 iterations. It can be seen from Fig. 17 that the difference between the quantity **curl**(*u*) ⋅ *n* and the desired curve ${B_{d}^{n}}$ inside the air gap decreases significantly.

### Surface PDEs

**Fig. 18:**
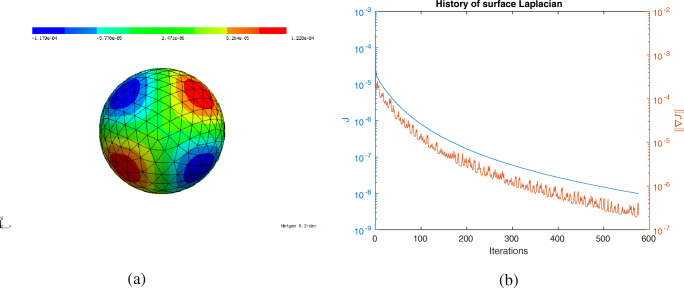
**a** Initial geometry for shape optimisation with respect to surface PDE (48–49). **b** History of objective value and norm of shape gradient using a first-order algorithm with line search

**Fig. 19:**
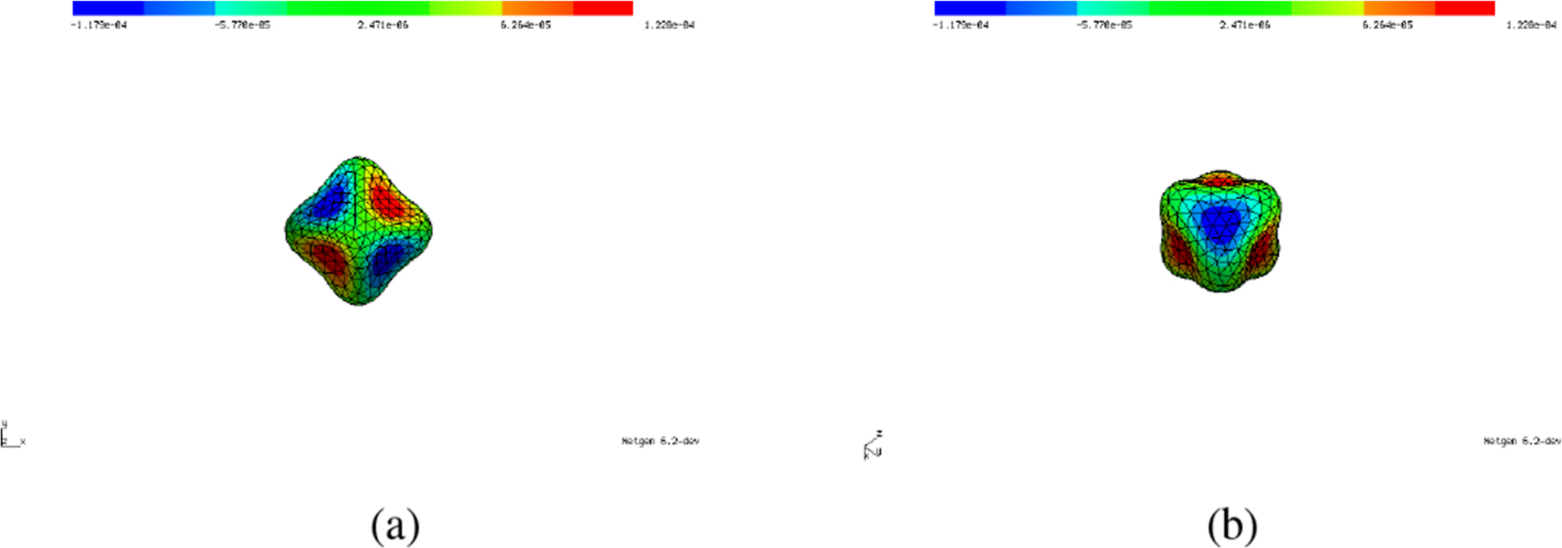
**a** Final design after 575 iterations. **b** Different view of **a**

Next, we also show the application of a shape optimisation algorithm to a problem constrained by a surface PDE. We solve problem (48–49) with *u*_*d*_ = 0, *f*(*x*_1_,*x*_2_,*x*_3_) = *x*_1_*x*_2_*x*_3_ and initial shape *M* = *S*^2^ the unit sphere in **R**^3^. We applied a first-order algorithm with a line search. Figure 18 shows the initial geometry as well as the decrease of the objective function and of the norm of the shape gradient. The objective value was reduced from 7.08 ⋅ 10^− 4^ to 9.88 ⋅ 10^− 9^. Figure 19 shows the final design which was obtained after 575 iterations from two different perspectives. The experiment was conducted using a surface mesh with 332 vertices and 660 faces and polynomial degree 3 (resulting in 2972 degrees of freedom).

### Time-dependent PDE using space-time method

In this section, we illustrate a non-standard situation where the fully automated shape differentiation using the command .DiffShape() fails, but the semi-automated way can be used to compute the shape derivative.

The situation is that of a parabolic PDE constraint in a space-time setting where the time variable is considered as just another space variable. Let *T* > 0 and *Ω* ⊂**R**^*d*^ be given and define the space-time cylinder *Q* :=*Ω* ×(0,*T*) ⊂**R**^*d*+ 1^. For given smooth functions *u*_*d*_ and *f* defined on *Q*, we consider the problem:

87 $$ \begin{array}{@{}rcl@{}} \underset{\varOmega}{\text{min }} &{\int}_{Q} |u-u_{d}|^{2}  \text d (x,\tau) \end{array} $$

88 $$ \begin{array}{@{}rcl@{}} \text{ s.t. } {\int}_{Q} \partial_{\tau} u v &+ \nabla_{x} u \cdot \nabla_{x} v  \text d(x,\tau) = {\int}_{Q} f v  \text d (x,\tau) \end{array} $$

for all *v* in the Bochner space $L^{2}(0,T; {H^{1}_{0}}(\varOmega ))$ where the state *u* is to be sought in the Bochner space $L^{2}(0,T; {H^{1}_{0}}(\varOmega )) \cap H^{1}(0,T; H^{-1}(\varOmega ))$ with the initial condition *u*(*x*,0) = 0. Here, $\nabla _{x} = (\partial _{x_{1}}, \dots , \partial _{x_{d}})^{\top }$ denotes the spatial gradient and *∂*_*τ*_ the time derivative. Note that we denote the time variable by *τ* in order not to interfere with the shape parameter *t*. We refer the interested reader to (Steinbach 2015) for details on this space-time formulation of the PDE constraint. As it is outlined there, the PDE can be solved numerically by choosing the same ansatz and test space consisting of piecewise linear and globally continuous finite element functions on *Q*.

For simplicity, we restrict ourselves to the case where *d* = 1, i.e. to the case where the spatial domain *Ω* is an interval. We are interested in the shape derivative of problem (87) with respect to spatial perturbations, i.e., with respect to transformations of the form:

$$ \begin{array}{@{}rcl@{}} T_{t}(x,\tau) = \left( \begin{array}{c} x + t V(x, \tau) \\ \tau \end{array} \right) \end{array} $$

where *V* ∈ *C*^0,1^(*Q*,**R**^*d*^) and *t* ≥ 0. We recall the notation *F*_*t*_(*x*,*τ*) = *∂T*_*t*_(*x*,*τ*). By this choice of transformation *T*_*t*_ we exclude an unwanted deformation of the space-time cylinder into the time direction as the time horizon *T* > 0 is assumed to be fixed.

Following the lines of the previous examples, we can define the cost function, the PDE and the Lagrangian: 
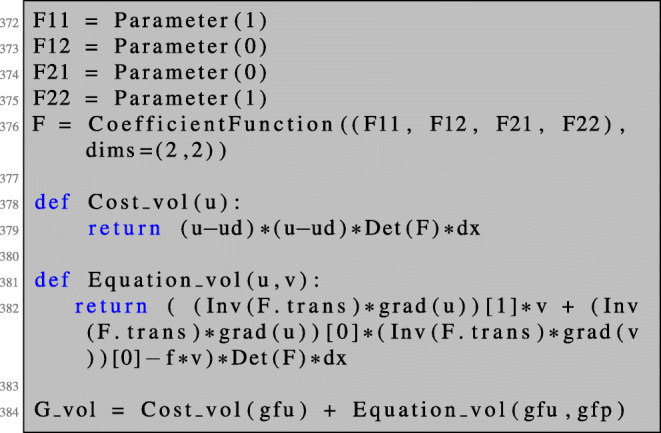


Here, gfu denotes the solution to the state (88) and gfp the solution to the adjoint equation, which is posed backward in time and reads in its strong form:

$$ \begin{array}{@{}rcl@{}} -\partial_{\tau} p - {\Delta} p &=& - 2(u-u_{d}) (x,\tau) \in Q, \\ p(x, \tau) &=& 0 (x,\tau) \in \partial \varOmega \times (0,T),\\ p(x,T) &=& 0 x \in \varOmega. \end{array} $$

We can compute the shape derivative similarly to the previous examples by means of formula (10), i.e.,

89 $$ \begin{array}{@{}rcl@{}} \left. \frac{d}{dt} \mathcal{J}(\varOmega_{t}) \right|_{t=0} &= \left. \left( \frac{dG}{dT_{t}} \frac{d T_{t}}{dt} + \frac{dG}{dF_{t}} \frac{d F_{t}}{dt}\right)\right|_{t=0}. \end{array} $$

However, it must be noted that in this special situation we have

90 $$ \begin{array}{@{}rcl@{}} \frac{d T_{t}}{dt} = \left( \begin{array}{c} V \\ 0 \end{array} \right)  \text{ and }  \frac{d F_{t}}{dt} = \left( \begin{array}{cc} \partial_{x} V & \partial_{\tau} V\\ 0&0 \end{array} \right). \end{array} $$

The shape derivative can now be obtained as follows: Given a mesh of the space-time cylinder *Q*, we define an **R**^*d*^-valued *H*^1^-space to represent the vector field *V* (here we assumed *d* = 1, thus we are facing a scalar-valued space). The shape derivative is a linear functional on this space and is obtained by plugging in (90) into (89): 
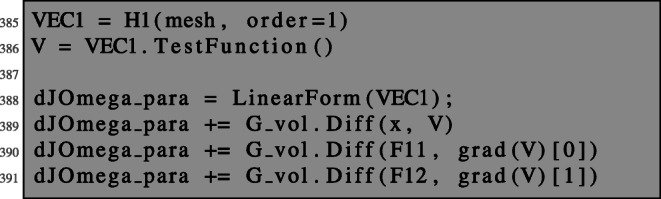


#### *Remark 7*

The fully automated shape differentiation command .DiffShape(V) cannot be used here because the vector field *V* has fewer components than the dimension of the mesh. On the other hand, if *V* was chosen as a vector field with *d* + 1 components, the command .DiffShape(V) would evaluate formula (89), but would assume $\frac {d T_{t}}{dt} = V = (V_{x}, V_{\tau })^{\top }$ and

$$\frac{d F_{t}}{dt} = \partial V = \left( \begin{array}{cc} \partial_{x} V_{x} & \partial_{\tau} V_{x}\\ \partial_{x} V_{\tau} & \partial_{\tau} V_{\tau} \end{array} \right)$$

and could not take into account the special situation at hand as shown in (90). This example is meant to illustrate the greater flexibility of the semi-automated compared with the fully automated shape differentiation.

Code lines 388–391 show the computation of the shape derivative in the direction of an **R**^*d*^-valued function *V* = *V* (*x*,*τ*) (recall *d* = 1 here). However, using *τ*-dependent vector fields would result in time-dependent optimal shapes, which is often not desired. Rather, one is interested in vector fields of the form *V* = *V* (*x*)≠*V* (*x*,*τ*) which still yield a descent, i.e. $D \mathcal {J}(\varOmega )(V) < 0$. This can be achieved as follows:

1. Compute a time-dependent shape gradient $\tilde W$ by solving
  91 $$ \begin{array}{@{}rcl@{}} {\int}_{Q} \partial \tilde W : \partial \tilde V + \tilde W \cdot \tilde V = D\mathcal{J}(\varOmega)(\tilde V) \quad \text{for all } \tilde V, \end{array} $$
2. Set $W(x, \tau ) = \frac {1}{T} {{\int \limits }_{0}^{T}} \tilde W(x, s) \text {ds}$.

Note that *W*(*x*,*τ*) is constant in *τ*. Then we see by plugging in $\tilde V = -W$ in (91) that

$$ \begin{array}{@{}rcl@{}} D \mathcal{J}(\varOmega)(-W) <0, \end{array} $$

thus − *W* is a descent direction.

We used this strategy to solve problem (87) for *d* = 1 with the data *u*_*d*_(*x*,*τ*) = *x*(1 − *x*)*τ*, *f*(*x*,*τ*) = *x*(1 − *x*) + 2*τ* numerically starting out from the initial domain *Ω*_*init*_ = (0.2,0.8) and the fixed time interval (0,*T*) = (0,1). Note that the data is chosen such that the domain *Ω*^⋆^ = (0,1) is a global solution to problem (87).

**Fig. 20:**
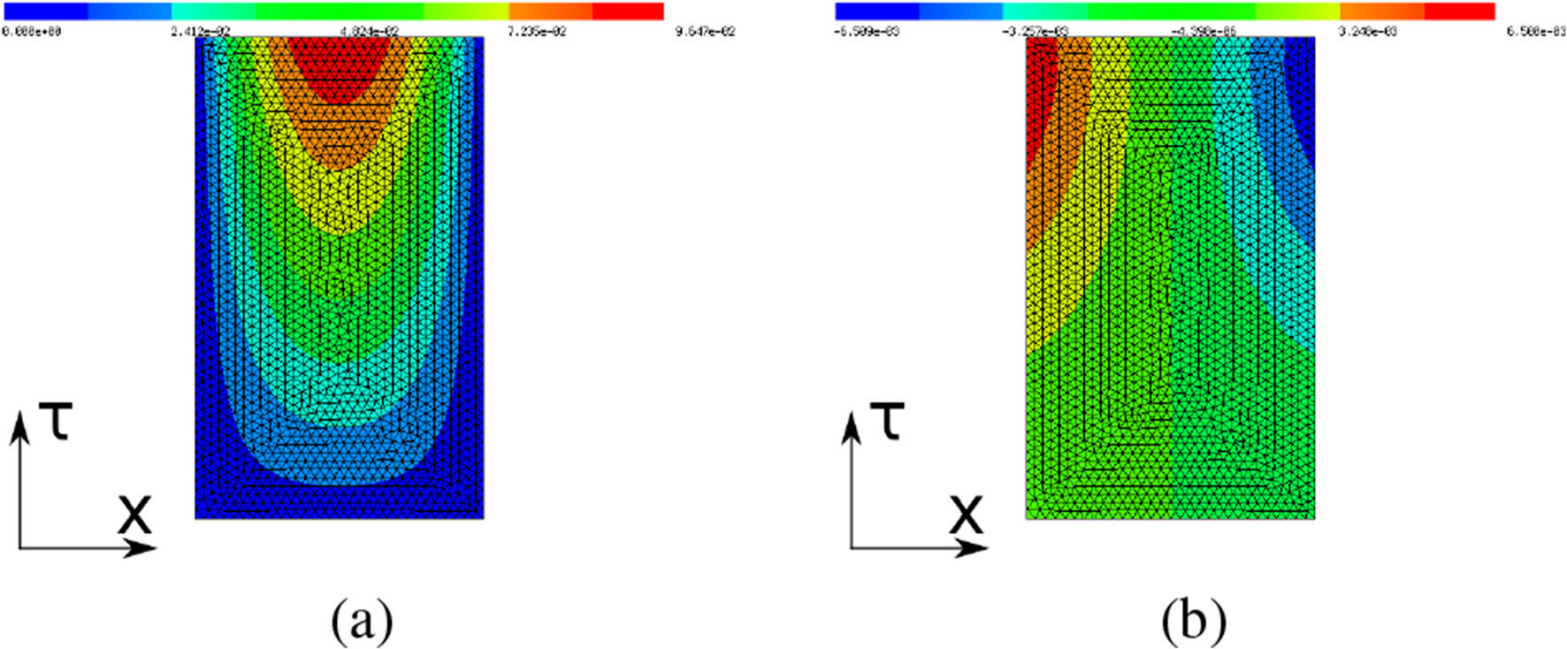
Space-time cylinder in initial configuration. **a** Solution to state (88). **b** Time-dependent descent vector field $\tilde W$ obtained by solving (91)

**Fig. 21:**
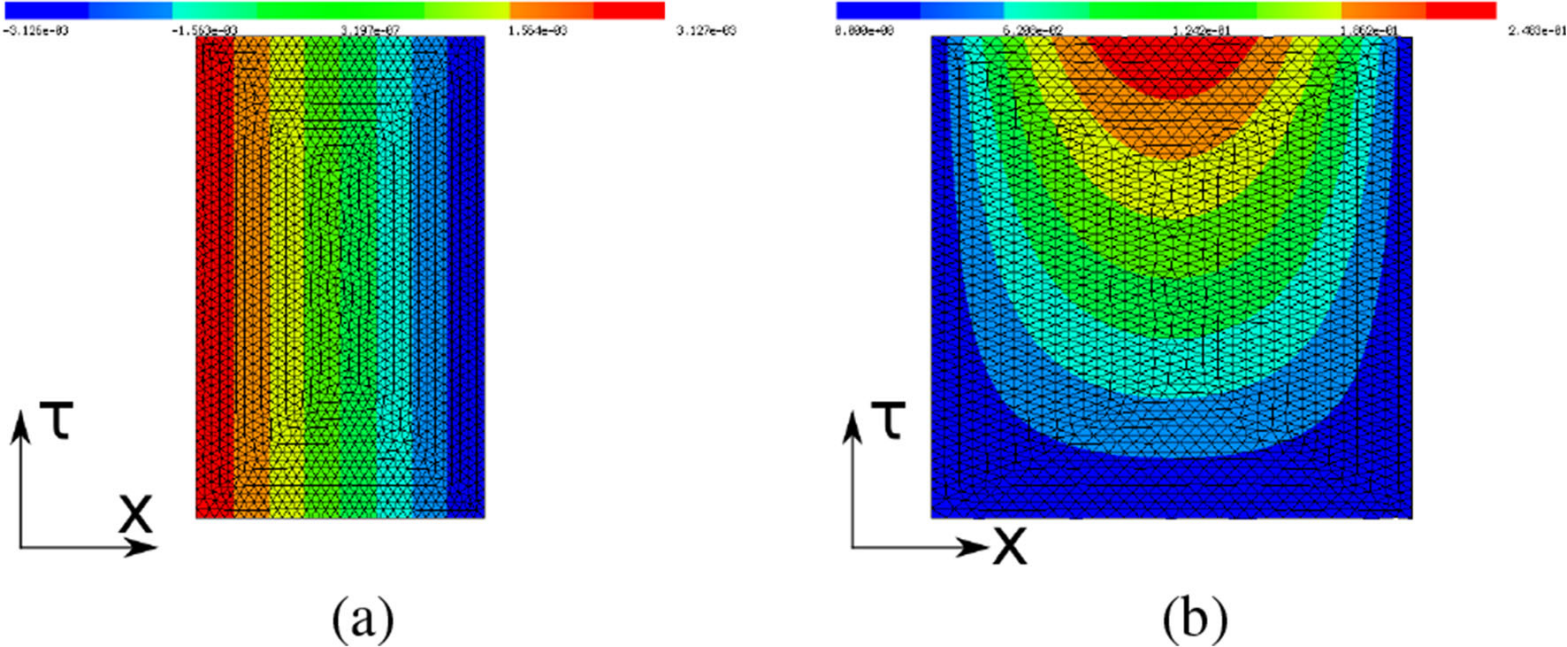
Space-time cylinder in initial and final configurations. **a** Time-independent descent vector field *W* obtained by averaging $\tilde W$. **b** Solution to state (88) on final design

Figure 20 shows the initial design together with the solution to the state equation and the time-dependent descent vector field $\tilde W$ obtained as solution of (91). Figure 21a shows the averaged vector field *W* which is independent of *τ* and yields a uniform deformation of the space-time cylinder. The final design after 293 iterations can be seen in Fig. 21b. The cost function was reduced from 4.65 ⋅ 10^− 3^ to 9.95 ⋅ 10^− 9^.

For more details on the implementation of this example, we refer to Online Resource 1 and for more details on shape optimisation in a space-time setting, we refer the interested reader to Köthe (2020).

## Conclusion

We showed how to obtain first- and second-order shape derivatives for unconstrained and PDE-constrained shape optimisation problems in a semi-automatic and fully automatic way in the finite element software package NGSolve. We verified the proposed method numerically by Taylor tests and by showing its successful application to several shape optimisation problems. We believe that this intuitive approach can help research scientists working in the field of shape optimisation to further improve numerical methods on the one hand, and product engineers working with NGSolve to design devices in an optimal fashion on the other hand.

## Electronic supplementary material

Below is the link to the electronic supplementary material.

(ZIP 42.4 KB)
